# Multi-site benchmark classification of major depressive disorder using machine learning on cortical and subcortical measures

**DOI:** 10.1038/s41598-023-47934-8

**Published:** 2024-01-11

**Authors:** Vladimir Belov, Tracy Erwin-Grabner, Moji Aghajani, Andre Aleman, Alyssa R. Amod, Zeynep Basgoze, Francesco Benedetti, Bianca Besteher, Robin Bülow, Christopher R. K. Ching, Colm G. Connolly, Kathryn Cullen, Christopher G. Davey, Danai Dima, Annemiek Dols, Jennifer W. Evans, Cynthia H. Y. Fu, Ali Saffet Gonul, Ian H. Gotlib, Hans J. Grabe, Nynke Groenewold, J Paul Hamilton, Ben J. Harrison, Tiffany C. Ho, Benson Mwangi, Natalia Jaworska, Neda Jahanshad, Bonnie Klimes-Dougan, Sheri-Michelle Koopowitz, Thomas Lancaster, Meng Li, David E. J. Linden, Frank P. MacMaster, David M. A. Mehler, Elisa Melloni, Bryon A. Mueller, Amar Ojha, Mardien L. Oudega, Brenda W. J. H. Penninx, Sara Poletti, Edith Pomarol-Clotet, Maria J. Portella, Elena Pozzi, Liesbeth Reneman, Matthew D. Sacchet, Philipp G. Sämann, Anouk Schrantee, Kang Sim, Jair C. Soares, Dan J. Stein, Sophia I. Thomopoulos, Aslihan Uyar-Demir, Nic J. A. van der Wee, Steven J. A. van der Werff, Henry Völzke, Sarah Whittle, Katharina Wittfeld, Margaret J. Wright, Mon-Ju Wu, Tony T. Yang, Carlos Zarate, Dick J. Veltman, Lianne Schmaal, Paul M. Thompson, Roberto Goya-Maldonado, the ENIGMA Major Depressive Disorder working group

**Affiliations:** 1 Laboratory of Systems Neuroscience and Imaging in Psychiatry (SNIP-Lab), Department of Psychiatry and Psychotherapy, University Medical Center Göttingen (UMG), Georg-August University, Von-Siebold-Str. 5, 37075 Göttingen, Germany; 2 Department of Psychiatry, Amsterdam UMC, Amsterdam Neuroscience, Amsterdam Public Health Research Institute, Vrije Universiteit Amsterdam, Amsterdam, The Netherlands; 3 Institute of Education and Child Studies, Section Forensic Family and Youth Care, Leiden University, Leiden, The Netherlands; 4 Department of Biomedical Sciences of Cells and Systems, University Medical Center Groningen, University of Groningen, Groningen, The Netherlands; 5 Department of Psychiatry and Mental Health, University of Cape Town, Cape Town, South Africa; 6 Department of Psychiatry and Behavioral Science, University of Minnesota Medical School, Minneapolis, MN USA; 7 Division of Neuroscience, IRCCS San Raffaele Scientific Institute, Milan, Italy; 8 Department of Psychiatry and Psychotherapy, Jena University Hospital, Jena, Germany; 9 Institute for Radiology and Neuroradiology, University Medicine Greifswald, Greifswald, Germany; 10 Imaging Genetics Center, Mark and Mary Stevens Neuroimaging and Informatics Institute, Keck School of Medicine, University of Southern California, Marina del Rey, CA USA; 11 Department of Biomedical Sciences, Florida State University, Tallahassee, FL USA; 12 Melbourne Neuropsychiatry Centre, Department of Psychiatry, The University of Melbourne, Parkville, VIC Australia; 13 Department of Psychology, School of Arts and Social Sciences, City, University of London, London, UK; 14 Department of Neuroimaging, Institute of Psychiatry, Psychology and Neuroscience, King’s College London, London, UK; 15 Experimental Therapeutics and Pathophysiology Branch, National Institute for Mental Health, National Institutes of Health, Bethesda, MD USA; 16 School of Psychology, University of East London, London, UK; 17 Centre for Affective Disorders, Institute of Psychiatry, Psychology and Neuroscience, King’s College London, London, UK; 18 SoCAT Lab, Department of Psychiatry, School of Medicine, Ege University, Izmir, Turkey; 19 Department of Psychology, Stanford University, Stanford, CA USA; 20 Department of Psychiatry and Psychotherapy, University Medicine Greifswald, Greifswald, Germany; 21 Center for Social and Affective Neuroscience, Department of Biomedical and Clinical Sciences, Linköping University, Linköping, Sweden; 22 Center for Medical Imaging and Visualization, Linköping University, Linköping, Sweden; 23 Department of Psychiatry and Behavioral Sciences, Division of Child and Adolescent Psychiatry, Weill Institute for Neurosciences, University of California, San Francisco, San Francisco, CA USA; 24 Department of Psychology, University of California, Los Angeles, Los Angeles, CA USA; 25 Louis A. Faillace, MD, Department of Psychiatry and Behavioral Sciences, The University of Texas Health Science Center at Houston, Houston, TX USA; 26 Center Of Excellence On Mood Disorders, Louis A. Faillace, MD, Department of Psychiatry and Behavioral Sciences at McGovern Medical School, The University of Texas Health Science Center at Houston, Houston, TX USA; 27 Department of Psychiatry, McGill University, Montreal, QC Canada; 28 Department of Psychology, University of Minnesota, Minneapolis, MN USA; 29 Cardiff University Brain Research Imaging Center, Cardiff University, Cardiff, UK; 30 MRC Center for Neuropsychiatric Genetics and Genomics, Cardiff University, Cardiff, UK; 31 Division of Psychological Medicine and Clinical Neurosciences, Cardiff University, Cardiff, UK; 32 School of Mental Health and Neuroscience, Faculty of Health, Medicine and Life Sciences, Maastricht University, Maastricht, The Netherlands; 33 Departments of Psychiatry and Pediatrics, University of Calgary, Calgary, AB Canada; 34 Department of Psychiatry, Psychotherapy and Psychosomatics, Medical School, RWTH Aachen University, Aachen, Germany; 35 Center for Neuroscience, University of Pittsburgh, Pittsburgh, PA USA; 36 Center for Neural Basis of Cognition, University of Pittsburgh, Pittsburgh, PA USA; 37 FIDMAG Germanes Hospitalàries Research Foundation, Centro de Investigación Biomédica en Red de Salud Mental (CIBERSAM), Barcelona, Catalonia Spain; 38 Sant Pau Mental Health Research Group, Institut de Recerca de L’Hospital de La Santa Creu I Sant Pau, Barcelona, Catalonia Spain; 39 Centre for Youth Mental Health, The University of Melbourne, Parkville, VIC Australia; 40 Orygen, Parkville, VIC Australia; 41 Department of Radiology and Nuclear Medicine, Amsterdam University Medical Centers, Amsterdam, The Netherlands; 42 Meditation Research Program, Department of Psychiatry, Massachusetts General Hospital, Harvard Medical School, Boston, MA USA; 43 Max Planck Institute of Psychiatry, Munich, Germany; 44 West Region, Institute of Mental Health, Singapore, Singapore; 45 Yong Loo Lin School of Medicine, National University of Singapore, Singapore, Singapore; 46 Lee Kong Chian School of Medicine, Nanyang Technological University, Singapore, Singapore; 47 SA MRC Research Unit on Risk and Resilience in Mental Disorders, Department of Psychiatry and Neuroscience Institute, University of Cape Town, Cape Town, South Africa; 48 Leiden Institute for Brain and Cognition, Leiden University Medical Center, Leiden, The Netherlands; 49 Department of Psychiatry, Leiden University Medical Center, Leiden, The Netherlands; 50 Institute for Community Medicine, University Medicine Greifswald, Greifswald, Germany; 51 Melbourne Neuropsychiatry Centre, Department of Psychiatry, The University of Melbourne and Melbourne Health, Melbourne, VIC Australia; 52 German Center for Neurodegenerative Diseases (DZNE), Site Rostock/ Greifswald, Greifswald, Germany; 53 Queensland Brain Institute, The University of Queensland, Brisbane, QLD Australia; 54 Centre for Advanced Imaging, The University of Queensland, Brisbane, QLD Australia; 55 Section on the Neurobiology and Treatment of Mood Disorders, National Institute of Mental Health, Bethesda, MD USA

**Keywords:** Diagnostic markers, Learning algorithms

## Abstract

Machine learning (ML) techniques have gained popularity in the neuroimaging field due to their potential for classifying neuropsychiatric disorders. However, the diagnostic predictive power of the existing algorithms has been limited by small sample sizes, lack of representativeness, data leakage, and/or overfitting. Here, we overcome these limitations with the largest multi-site sample size to date (N = 5365) to provide a generalizable ML classification benchmark of major depressive disorder (MDD) using shallow linear and non-linear models. Leveraging brain measures from standardized ENIGMA analysis pipelines in FreeSurfer, we were able to classify MDD versus healthy controls (HC) with a balanced accuracy of around 62%. But after harmonizing the data, e.g., using ComBat, the balanced accuracy dropped to approximately 52%. Accuracy results close to random chance levels were also observed in stratified groups according to age of onset, antidepressant use, number of episodes and sex. Future studies incorporating higher dimensional brain imaging/phenotype features, and/or using more advanced machine and deep learning methods may yield more encouraging prospects.

## Introduction

Major depressive disorder (MDD) is a psychiatric disorder with great impact on society, with a lifetime prevalence of 14%^1^, often resulting in reduced quality of life^2^ and increased risk of suicide for those affected^3^. Considering the possibility of treatment resistance^4^ and accelerated brain aging^5^, early recognition and implementation of effective treatments are critical. Unfortunately, there are no reliable biomarkers to date to diagnose MDD, to predict its highly variable natural progression or response to treatment^6^. Until now, the diagnosis of MDD relies exclusively on self-reported symptoms in clinical interviews, which—despite great efforts—present risk of misdiagnosis due to subjectivity and limited specificity of some symptoms, especially in the early stage of mental disorders. Furthermore, comorbid conditions such as substance use disorders, anxiety spectrum disorders^7^, and other mental and somatic diseases^8^ may contribute to the difficulty of correctly diagnosing and treating MDD.

With modern neuroimaging techniques such as magnetic resonance imaging (MRI), it has become possible to investigate cortical and subcortical brain alterations associated with MDD with high spatial resolution. Numerous studies reveal structural brain differences in MDD compared to healthy controls (HC)^9–13^, with patients presenting, on average, smaller hippocampal volumes as well as lower cortical thickness in the insula, temporal lobes, and orbitofrontal areas. However, inference at the group level and small effect sizes preclude clinical application. Analytic tools such as machine learning (ML) that allow multivariate combinations of brain features and enable inference at the individual level may result in better discrimination between MDD patients and HC, thereby potentially providing clinically relevant biomarkers for MDD.

Current literature shows MRI-based MDD classification accuracies ranging from 53 to 91%^14,15^ with inconsistencies regarding which brain regions are the most informative for the classification. This lack of consensus in the literature raises concerns regarding the generalizability of the classification methods and their related findings. A major contributor to high variability in classification performances is sample size^16,17^. Specifically smaller samples of the test data set tend to show more extreme classification accuracies in both directions from chance level^16^, whereas studies with larger sample sizes in the test set tend to converge to an accuracy of around 60%^17^. In the presence of publication bias, which favors the reporting of overestimations, published literature can quickly accumulate inflated results^18^. Further, overestimations in the neuroimaging field may also be driven by data leakage, which refers to the use of any information from the test set in any part of the training process^19,20^.

Another factor contributing to inconsistencies in results is the heterogeneity of samples in relation to demographic and clinical characteristics, which plays a significant role both in MDD and in the general population^5,21,22^. As large representative samples within a single cohort is difficult (e.g., due to financial cost, access to patient population, etc.), there is a growing interest in performing multi-site mega-analyses to address these issues.

ENIGMA MDD is a large-scale worldwide consortium, which curates and applies standardized analysis protocols to MRI and clinical/demographic data of MDD patients and HC from 52 independent sites from 17 countries across 6 continents (for review^23^,). Such large-scale approaches with global representation are necessary for identifying brain alterations associated with MDD that are realistic, reliable, and generalizable^24^. Therefore, we consider data from different international cohorts included in ENIGMA MDD a powerful and efficient resource to benchmark the robustness of representative examples of shallow linear and non-linear ML algorithms. Such algorithms include support vector machines (SVM), logistic regression with least absolute shrinkage and selection operator (LASSO) and ridge regularization, elastic net, and random forests. An additional advantage of ENIGMA MDD is that the inclusion of thousands of participants allows the stratification of several important factors related to cortical and subcortical brain alterations in MDD such as sex, age of MDD onset, number of depressive episodes, and antidepressant use. However, unifying multi-site data presents challenges. The global group differences between cohorts—referred to here as a site effect—may arise from different MR acquisition equipment and acquisition protocols^25^, and/or demographic and clinical factors^26,27^. Ignoring the site effect may lead to construction of suboptimal less-generalizable classification models^28^, hindering the generalizability of the results. Along these lines, a commonly used strategy to mitigate site effect is to apply a harmonization technique such as ComBat^29^. Adopted from genomic studies, NeuroComBat estimates and statistically corrects for (harmonizes) differences in location (mean) and scale (variance) across different cohorts, while preserving or perhaps even enhancing the effect size of the variables of interest^30–32^. There are only a few studies attempting large sample multi-site MDD classification using structural brain metrics^16,17^; however, site effects were not addressed in their analyses.

The main goal of this study was to establish a benchmark for classification of MDD versus HC based on structural cortical and subcortical brain measures in the largest sample to date. We profiled the classification performance of representative examples of linear and shallow non-linear models, including SVM with linear and rbf kernels with and without feature selection (PCA, t-test), logistic regression with LASSO/ridge regularization, elastic net and random forest. The model’s performance is estimated via balanced accuracy, area under the receiver operating characteristic (AUC), sensitivity and specificity. We hypothesized that all models would be able to classify MDD versus HC with balanced accuracy higher than random chance, based on provided brain measures. We pooled preprocessed structural data from ENIGMA MDD participants, including 5365 subjects (2288 MDD and 3077 HC) from 30 cohorts worldwide. As we were equally interested in general structural brain alterations in MDD as well as the generalizability of classification performance in sites unseen in the training phase, the data were split according to two strategies. First, age and sex (Splitting by Age/Sex) were evenly distributed across all cross-validation (CV) folds, where each fold is used as a test set once and the rest of folds is used as a training set iteratively. Second, the sites (Splitting by Site) were kept whole across CV folds, so the algorithms were trained and tested on different sets of cohorts, resulting in large between-sample heterogeneity of training and test sets, potentially resulting in lower classification performance^33^, especially if large site effects are present. Because MDD is a highly heterogeneous diagnosis—and previous work from ENIGMA MDD^10,11^ has identified distinct alterations in different clinical subgroups—we also stratified MDD based on sex, age of onset, antidepressant use, and number of depressive episodes to investigate whether classification accuracy could be improved when considering more homogenous subgroups. Additionally, we investigated which brain areas were most relevant to classification performance.

In summary, we expected that (1) All models would correctly classify MDD above chance level, (2) Splitting by Site would yield lower performance versus Splitting by Age/Sex, (3) Application of ComBat would improve classification performance for all models, and (4) Stratified analyses according to demographic and clinical characteristics would yield higher classification performance. We also explored the impact of other approaches to remove site effects (ComBat-GAM^34^ and CovBat^35^) from structural brain measures prior to feeding these measures into the classification models.

## Results

### Participants and data splitting

From 5572 participants, 207 were excluded due to less than 75% of combined cortical and subcortical features being provided, resulting in 5365 subjects (2288 MDD and 3077 HC) used in the analysis.

Substantial differences in age (87% of pairwise comparisons between cohorts were significant, t-test, *p* < 0.05) and sex (54%, t-test, *p* < 0.05) distribution exist in the investigated cohorts (Table 1, Supplementary Table 4). In the Splitting by Age/Sex strategy, all cohorts were evenly distributed across the folds, resulting in a similar number of subjects in each of fold (Table 2 left). In the Splitting by Site strategy, entire cohorts were kept into single folds, this time balancing the total number of subjects in each fold as close as possible (Table 2 right). This resulted in an irregular number of participants in each fold, with some folds containing only one of the larger cohorts (e.g., SHIP-T0, SHIP-S2, MPIP) and others containing multiple smaller cohorts.

**Table 1:** ENIGMA MDD participating cohorts in the study. Each cohort is presented with number of total subjects, number of patients with major depressive disorder (MDD) and healthy controls (HC), as well as their mean age (in years) and sex (number and % of females).

Cohort	Number of subjects	Age mean (SD)	Number of females (%)
AFFDIS			
Total	79	39.75 (14.67)	36 (46)
HC	46	39.87 (14.29)	22 (48)
MDD	33	39.58 (15.18)	14 (42)
Pharmo (AMC)			
Total	51	29.37 (4.64)	51 (100)
HC	0	N/A	N/A
MDD	51	29.37 (4.64)	51 (100)
Barcelona-StPau			
Total	94	46.66 (7.81)	72 (77)
HC	32	46.03 (8.00)	23 (72)
MDD	62	46.98 (7.68)	49 (79)
CARDIFF			
Total	40	46.55 (11.74)	27 (68)
HC	0	N/A	N/A
MDD	40	46.55 (11.74)	27 (68)
CSAN			
Total	109	34.70 (12.88)	74 (68)
HC	49	33.20 (12.07)	34 (69)
MDD	60	35.92 (13.38)	40 (67)
Calgary			
Total	107	17.03 (4.12)	60 (56)
HC	52	15.81 (5.03)	29 (56)
MDD	55	18.19 (2.51)	31 (56)
DCHS			
Total	79	30.91 (6.71)	79 (100)
HC	61	31.49 (6.82)	61 (100)
MDD	18	28.94 (5.89)	18 (100)
ETPB			
Total	60	35.03 (9.86)	36 (60)
HC	26	33.88 (10.22)	16 (62)
MDD	34	35.91 (9.48)	20 (59)
Episca (Leiden)			
Total	49	15.00 (1.54)	42 (86)
HC	30	14.73 (1.53)	26 (87)
MDD	19	15.42(1.46)	16 (84)
FIDMAG			
Total	69	47.22 (12.29)	44 (64)
HC	34	45.94 (11.49)	22 (65)
MDD	35	48.46 (12.90)	22 (63)
Groningen			
Total	41	44.27 (13.67)	30 (73)
HC	21	44.05 (13.96)	16 (76)
MDD	20	44.50 (13.34)	14 (70)
Houston			
Total	290	28.72 (16.30)	169 (58)
HC	186	26.76 (15.91)	105 (56)
MDD	104	32.23 (16.39)	64 (62)
Jena			
Total	107	46.76 (15.00)	52 (49)
HC	77	47.75 (15.93)	36 (47)
MDD	30	44.20 (11.92)	16 (53)
LOND			
Total	130	49.67 (8.62)	79 (61)
HC	61	51.72(7.87)	32 (53)
MDD	69	47.86(8.85)	47 (68)
MODECT			
Total	42	72.71 (9.25)	28 (67)
HC	0	N/A	N/A
MDD	42	72.71 (9.25)	28 (67)
MPIP			
Total	548	48.66 (13.59)	315 (57)
HC	211	49.53 (13.02)	124 (59)
MDD	337	48.12 (13.90)	191 (57)
Melbourne			
Total	245	19.42 (2.88)	130 (53)
HC	102	19.58 (2.97)	54 (53)
MDD	143	13.31 (2.80)	76 (53)
Minnesota			
Total	110	15.47 (1.89)	79 (72)
HC	40	15.68 (1.98)	26 (65)
MDD	70	15.36 (1.83)	53 (76)
Moraldilemma			
Total	70	18.81 (1.94)	70 (100)
HC	46	18.50 (1.75)	46 (100)
MDD	24	19.42 (2.14)	24 (100)
NESDA			
Total	219	38.11 (10.32)	145 (66)
HC	65	40.29 (9.67)	42 (65)
MDD	154	37.19 (10.45)	103 (67)
QTIM			
Total	386	22.08 (3.25)	267 (69)
HC	284	22.11 (3.30)	190 (67)
MDD	102	22.01 (3.11)	77 (75)
UCSF			
Total	163	15.46 (1.31)	91 (56)
HC	88	15.32 (1.28)	42 (48)
MDD	75	15.63 (1.33)	49 (65)
SHIP_S2			
Total	579	55.01 (12.57)	294 (51)
HC	443	55.44 (12.80)	198 (45)
MDD	136	53.59 (11.68)	96 (71)
SHIP_T0			
Total	1229	50.15 (13.69)	607 (49)
HC	919	50.50 (14.18)	405 (44)
MDD	310	49.12 (12.04)	202 (65)
SanRaffaele			
Total	45	49.07 (13.51)	32 (71)
HC	0	N/A	N/A
MDD	45	49.07 (13.51)	32 (71)
Singapore			
Total	38	39.50 (6.43)	18 (47)
HC	16	38.69 (4.59)	8(50)
MDD	22	40.09 (7.43)	10 (45)
Socat_dep			
Total	179	37.85 (13.34)	161 (90)
HC	100	36.42 (13.57)	90 (90)
MDD	79	39.66 (12.81)	71 (90)
StanfFAA			
Total	32	32.71 (9.56)	32 (100)
HC	18	30.44 (9.96)	18 (100)
MDD	14	35.63 (8.14)	14 (100)
StanfT1wAggr			
Total	115	37.18 (10.27)	69 (60)
HC	59	37.24 (10.43)	36 (61)
MDD	56	37.11 (10.09)	33 (59)
TIGER			
Total	60	15.63 (1.34)	38 (63)
HC	11	15.18 (1.03)	5 (45)
MDD	49	15.73 (1.38)	33 (67)
**All sites**			
Total	5365	39.84 (17.69)	3227 (60)
HC	3077	40.82(18.09)	1706 (55)
MDD	2288	38.52 (17.05)	1521 (66)

**Table 2:** Data splitting strategies. The differences in strategies are manifested in the distribution of age, sex, and diagnosis between cross-validation folds.

Splitting by Age/Sex				Splitting by Site			
Fold	Age mean (SD)	Number of females (%)	Number of subjects (%MDD)	Fold	Age mean (SD)	Number of females (%)	Number of subjects (%MDD)
1	39.98 (17.40)	322 (60)	536 (42)	1	50.15 (13.69)	607 (49)	1229 (25)
2	39.63 (17.81)	324 (60)	538 (42)	2	55.01 (12.57)	294 (51)	579 (23)
3	39.85 (17.57)	325 (60)	538 (43)	3	48.66 (13.59)	315 (57)	548 (61)
4	39.66 (17.94)	322 (60)	535 (39)	4	22.90 (4.97)	299 (72)	418 (28)
5	39.99 (17.56)	323 (60)	538 (44)	5	36.72 (19.69)	272 (60)	451 (51)
6	39.75 (17.25)	317 (60)	531 (43)	6	22.53 (10.92)	293 (65)	450 (68)
7	40.15 (17.89)	327 (60)	541 (42)	7	35.94 (12.96)	295 (71)	418 (59)
8	39.81 (17.93)	322 (60)	535 (44)	8	38.85 (12.66)	348 (81)	431 (45)
9	39.86 (17.73)	320 (60)	535 (44)	9	24.79 (16.16)	203 (54)	377 (42)
10	39.74 (17.80)	325 (60)	538 (43)	10	34.95 (15.45)	301 (65)	464 (55)

### Full data set analysis

The classification performance of all models was similar and is presented in Table 3. When sites were evenly distributed across all CV folds (Splitting by Age/Sex), the highest balanced accuracy of 0.639 was achieved by SVM with rbf kernel, when trained using all cortical and subcortical features. The application of ComBat harmonization resulted in a performance drop of all models close to chance level. This pattern of lower classification performance, when ComBat was applied, was also observed across other classification metrics (see Supplementary Tables 5, 6, 7). Yet specificity was found to be up to 10% higher than sensitivity, possibly related to potential imbalances in ratio MDD to HC and its effect on the classification. For the Splitting by Site strategy, classification performances did not change significantly based on whether the ComBat harmonization was performed or not. Balanced accuracy was close to random chance, indicating that the models were not able to differentiate MDD subjects from HC. The application of ComBat did not result in higher classification accuracies (Table 3). By exploring the classification performances measured on only a subset of cortical and subcortical features, we observed very similar results with classification around chance level. Similarly, there was no improvement when more sophisticated harmonization algorithms such as ComBat-GAM and CovBat were applied (see Supplementary Table 8).

**Table 3:** Balanced accuracy measured on the entire data set, after being divided into cross-validation folds using the Splitting by Age/Sex and Splitting by Site strategies. We evaluated classification performances when models are trained on combined cortical and subcortical features, cortical thickness, cortical surface area, and subcortical volume. Furthermore, all models were trained/tested without and with ComBat harmonization.

	Cortical + subcortical		Cortical thickness		Cortical surface area		Subcortical volume	
	No ComBat	With ComBat	No ComBat	With ComBat	No ComBat	With ComBat	No ComBat	With ComBat
**Splitting by Age/Sex**								
SVM linear	0.616	0.524	0.577	0.504	0.572	0.518	0.602	0.524
SVM rbf	0.639	0.525	0.600	0.515	0.578	0.510	0.619	0.513
SVM + PCA	0.638	0.529	0.601	0.513	0.575	0.518	0.622	0.513
SVM + ttest	0.627	0.515	0.581	0.515	0.567	0.526	0.619	0.521
LASSO	0.612	0.524	0.583	0.499	0.578	0.516	0.596	0.518
Ridge	0.610	0.523	0.585	0.498	0.573	0.515	0.594	0.520
Elastic Net	0.609	0.523	0.584	0.500	0.569	0.517	0.593	0.520
Random Forest	0.613	0.507	0.593	0.514	0.574	0.509	0.611	0.511
**Splitting by site**								
SVM linear	0.512	0.519	0.498	0.495	0.499	0.506	0.506	0.521
SVM rbf	0.513	0.515	0.493	0.499	0.493	0.513	0.503	0.519
SVM + PCA	0.527	0.520	0.502	0.512	0.504	0.524	0.520	0.520
SVM + ttest	0.502	0.512	0.487	0.499	0.507	0.508	0.510	0.527
LASSO	0.513	0.517	0.491	0.489	0.508	0.513	0.507	0.512
Ridge	0.514	0.514	0.497	0.490	0.505	0.509	0.507	0.514
Elastic Net	0.513	0.514	0.498	0.489	0.503	0.514	0.507	0.514
Random Forest	0.518	0.506	0.495	0.501	0.491	0.503	0.519	0.501

When no harmonization step was applied, the choice of CV splitting strategy affected all measures of classification performance. Splitting by Age/Sex strategy yielded a balanced accuracy above 0.60 compared to roughly 0.51 accuracy for the Splitting by Site strategy. The ComBat harmonization step evened the classification performance of algorithms for the different splitting strategies, both being close to random chance. Information on the balanced accuracy changes via ComBat performing leave-one-site-out CV, can be found in Supplementary Table 9.

**Figure 1:**
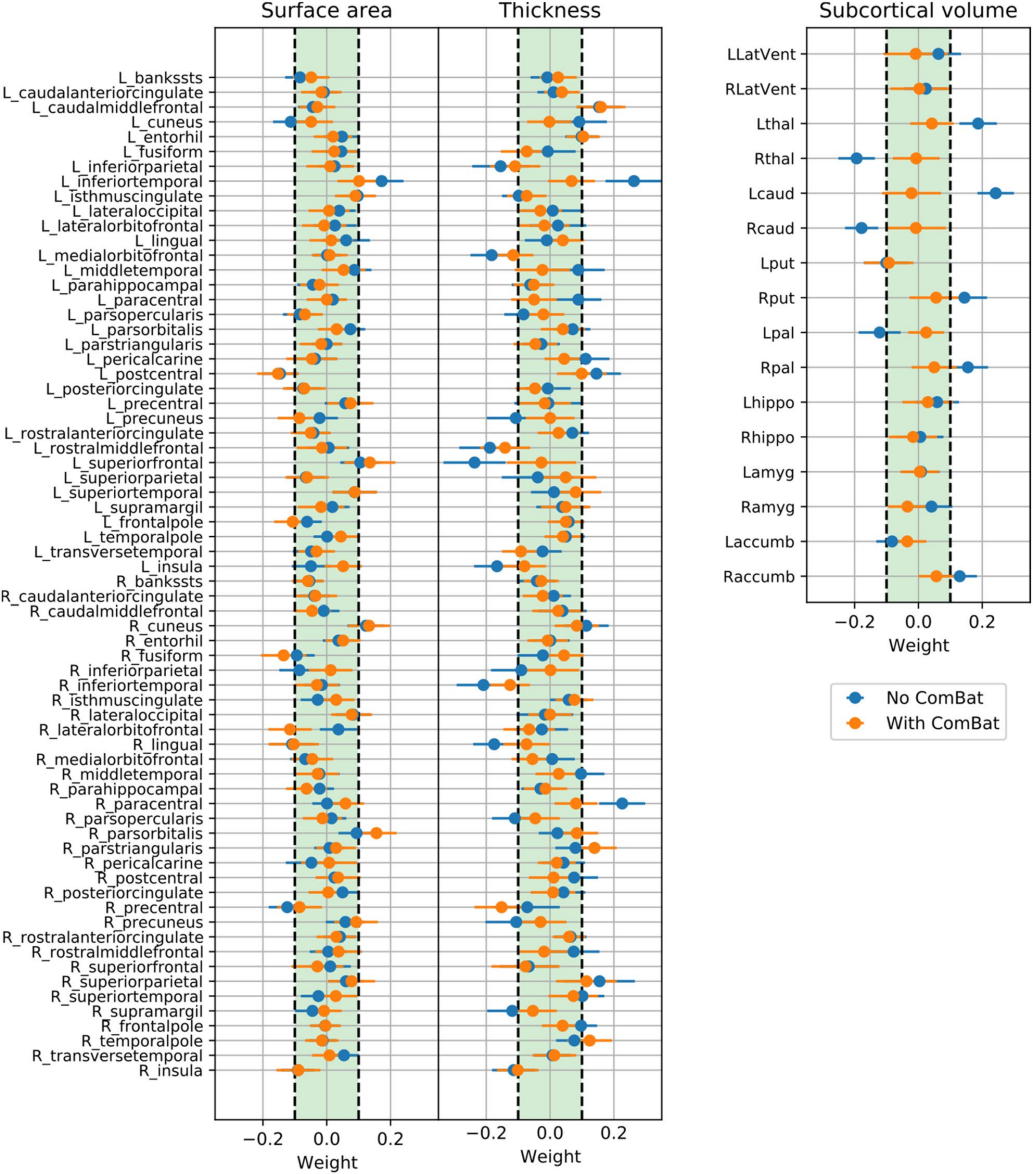
Feature weights of support vector machines (SVM) with the linear kernel. To assess the decision-making of SVM to differentiate subjects with major depressive disorder (MDD) from healthy controls (HC), we investigate the importance of the structural brain features by looking at the corresponding feature weights for the regional cortical surface areas, cortical thicknesses and subcortical volumes. The horizontal bars indicate the 95% confidence interval calculated using percentile method via bootstrapping.

**Figure 2:**
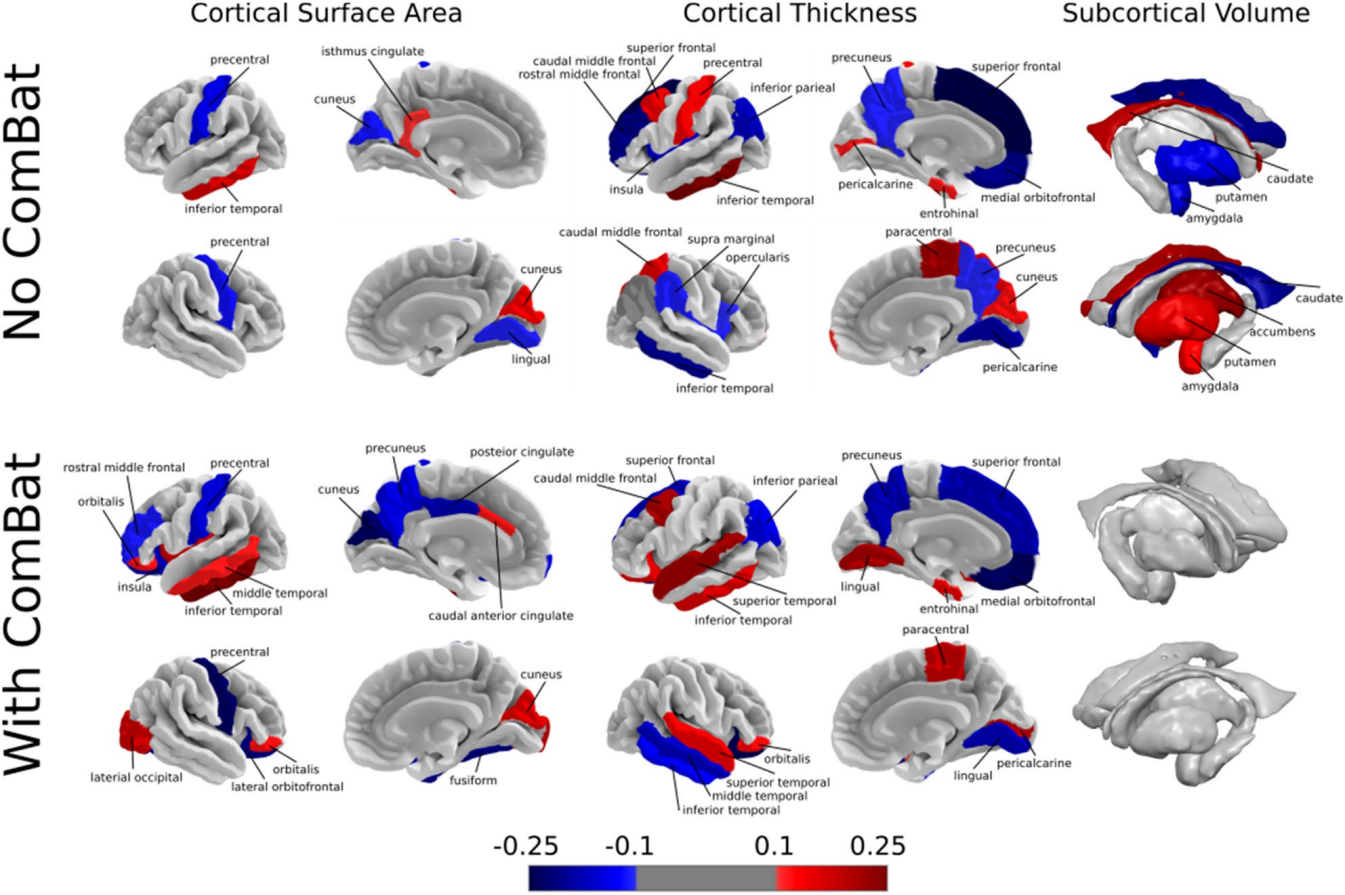
The most informative features for classification including regional cortical surface areas, thicknesses and subcortical volumes, trained on the whole data set without and with ComBat harmonization. Increased and decreased feature weight values in the major depressive disorder (MDD) group are represented by red and blue colormap, respectively.

As the performance of the models were similar across all conditions, we assessed the weights of SVM with linear kernel to investigate, which regions contributed the most to the classification. The performance of SVM with and without application of ComBat was primarily driven by roughly the same set of cortical features, which could be observed by examining the feature weights. Feature weights of the SVM with linear kernel are presented in Figs. 1 and 2. Even though the harmonization step affected the weights of the features, most of the informative features (with absolute weight > 0.1) remained present. Cortical thickness features had greater weights compared to cortical surface areas, among which the left caudal middle frontal, left inferior parietal, left and right inferior temporal, left medial orbitofrontal, left postcentral, left precuneus, left superior frontal, right lingual, right paracentral, and right superior temporal regions were informative with and without the harmonization step. In the case of the regional surface areas, left and right cuneus, left inferior temporal, left medial orbitofrontal, left postcentral, and right precentral regions were found to be most informative for classification. Among subcortical volumes, no features remained informative after removing site effect via ComBat.

### Data stratification

Next, we investigated the classification performance of models trained and tested on stratified data by demographic and clinical characteristics. The general pattern of the highest accuracy achieved by Splitting by Age/Sex strategy without ComBat and the significant drop in the accuracy when ComBat is applied was observed in all stratified analyses (below). In the Splitting by Site strategy, the classification performance did not change significantly when ComBat was applied. Information on the feature weights may be found in Supplementary Figs. 1, 2, 3, 4.

### Males versus females

The number of male subjects is 2131 and female subjects is 3227 (7 male participants from the Episca cohort were not considered as we could not split them into 10 CV folds). In the Splitting by Age/Sex strategy without the harmonization step, the highest balanced accuracy of 0.632 was achieved when trained and tested on males—compared to maximum of 0.585 for females. When ComBat was applied, the accuracy dropped to 0.530 for males and to 0.529 for females, showing that there were minimal differences in classification results for males and females. For Splitting by Site, the accuracy did not change depending on the use of ComBat for both males (0.513–0.506) and females (0.519–0.517). Nevertheless, different brain regions were found important for classification in subgroups. In general, more features were found to be important for classification for males compared to females; this is especially noticeable for the regional surface areas (Supplementary Fig. 1).

### Age of onset

For Splitting by Age/Sex, when only patients first diagnosed in adolescence were included in the analysis, yielding 3,794 subjects in total, an accuracy of 0.626 was achieved, compared to 0.623 when patients who were first diagnosed in adulthood were analyzed. These accuracies dropped to 0.548 and 0.521 respectively, when ComBat was applied. In the Splitting by Site strategy, the balanced accuracy metrics did not change substantially for both subgroups: 0.541 to 0.544 for the adolescent-onset group and 0.546–0.518 for the adult-onset group, highlighting the absence of significant differences between these groups (Supplementary Fig. 2).

### Antidepressant use versus antidepressant free (at the time of MR scan)

Both subgroups showed a drop in balanced accuracy when ComBat was applied. In case of Splitting by Age/Sex, it reduced from 0.564 to 0.529 for the antidepressant-free subgroup (4408 subjects) and from 0.716 to 0.534 for the antidepressant subgroup (3988 subjects). When Splitting by Site, the balanced accuracy metrics did not change significantly for any of the subgroups when ComBat was used. For the antidepressant-free subgroup, it decreased from 0.564 to 0.528, while for the antidepressant group, it dropped from 0.560 to 0.483 (Supplementary Fig. 3).

### First episode versus recurrent episodes

Similarly, a drop in accuracy was observed when the data set was stratified based on the number of depressive episodes with versus without ComBat. In Splitting by Age/Sex, the balanced accuracy for the first episode subgroup dropped from 0.559 to 0.518 when ComBat was applied. For individuals with more than one episode, the balanced accuracy decreased from 0.644 to 0.520 with ComBat. In the Splitting by Site strategy, the algorithm's performance was not majorly affected by ComBat in the single episode subgroup, yielding 0.482 to 0.512 in balanced accuracy and an insignificant drop from 0.521 to 0.505 for the recurrent episodes subgroup (Supplementary Fig. 4).

## Discussion

In this work, we benchmarked ML performance on the largest multi-site data set to date, using regional cortical and subcortical structural information for the task of discriminating patients with MDD versus HC. We applied shallow linear and non-linear models to 152 atlas-based features of 5365 subjects from the ENIGMA MDD working group. To investigate brain characteristics common to MDD, as well as realistic classification metrics for unseen sites, we used two different data splitting approaches. Balanced accuracy was up to 63%, when data was split into folds according to *Splitting by Age/Sex*, and up to 51%, when data was split into folds according to *Splitting by Site* strategy. The harmonization of the data via ComBat evened the classification performance for both data splitting strategies, yielding up to 52% of balanced accuracy. This classification level implies that initial differences in performances were due to the site effects, most likely stemming from differences in MRI acquisition protocols across sites. Lastly, the data set was stratified based on demographic and clinical factors, but we found only minor differences in terms of classification performances between subgroups.

### Data splitting and site effect

Splitting of the data plays an important role in formulating and testing the hypotheses as well as validating them. As shown in^36^, different data splitting techniques in combination with machine and deep learning algorithms in medical mega-analytical studies may introduce unwanted biases influencing classification or regression performances. Here we aimed to consider two data splitting paradigms: Splitting by Age/Sex and Splitting by Site. With Splitting by Age/Sex, we investigated general MDD alterations in contrast to HC using ML methods to obtain unbiased results regarding these important demographic factors. When we look at the weights of the SVM with linear kernel estimated on the entire data set, they correspond to the performance from Splitting by Age/Sex, as every CV fold contains all sites and demographically corresponds closely to the whole data set. With Splitting by Site, we wanted to see if the knowledge learned in one subset of cohorts could be translated to unseen cohorts—this can only be realistically measured when data is split according to the site it belongs to. *To the best of our knowledge, this is the first study to systematically emphasize differences in MDD versus HC classification performance in the context of data splitting strategies and the impact of ComBat in these strategies.* The balanced accuracy of algorithms trained on data from Splitting by Age/Sex was up to 10% higher compared to Splitting by Site, confirming our expectations. This is a common trend in multi-site neuroimaging analyses^37^, which indicates site effect and emphasizes how the nuances in data splitting strategies can strongly influence the classification performance. The presence of the site effect was additionally confirmed by training the SVM model to classify subjects according to their respective site, yielding substantially higher balanced accuracy compared to the main task of MDD versus HC classification (see Supplementary section “Harmonization methods”). The possibility that the site effect still reflected the demographic differences across cohorts, as cortical and subcortical features undergo substantial changes throughout lifespan^34^ and differ between males and females^21,22^, was not supported. Regressing out these sources of demographic information did not significantly change the level of classification when predicting site belonging. According to our results, a major source of the site effect comes from the different scanner models and acquisition protocols, since we achieved the highest accuracy when attempting to classify scanner type (see Suppl. “Harmonization methods”).

In addition to scanning differences, demographic and diagnostic characteristics distributions were different across the sites. Therefore, we explored if balancing the sample in terms of age and sex distributions would lead to higher classification performance. However, balancing of age/sex distributions across sites did not improve classification performance in Splitting by Site (balanced accuracy 52.6%/50.7% without/with ComBat). Thus, balancing age and sex did not contributed to better performance. As the MDD/HC ratio also varied across sites, an influence of site affiliation to the main MDD versus HC task could exist. Therefore, we additionally explored if the classification performance would drop to random level by equalizing the MDD/HC ratio in every site before splitting the data according to Splitting by Age/Sex. Sites without HC were discarded from this analysis. Indeed, we observed a substantial drop of the balanced accuracy from 61 to 53% with 1:1 MDD to HC ratio, confirming our assumption of likely incorporation of the site affiliation in the diagnosis classification.

Building on this, ComBat was able to remove the site effect, as all classification models could not differentiate between sites after its application. Subsequently, there were no differences between classification results across splitting approaches, with around 0.52 in balanced accuracy. Such a low accuracy—close to random chance—is consistent with another large sample study based on two cohorts^17^. In their study, self-reported current depression was speculated as a reason for low accuracy, but this possibility is unlikely explaining our classification results. Moreover, similar classification levels in our and their study support the notion that a more balanced ratio between classes is not the main aspect behind the low power of discrimination. Furthermore, single site classification analysis revealed 0.50 to 0.55 accuracy range for bigger cohorts, while smaller cohorts yielded wider range of classification results (Supplementary Table 10), in line with previous large sample study^16^.

Similar to ComBat, other more sophisticated harmonization methods such as ComBat-GAM and CovBat were able to remove site effect, but did not improve the balanced accuracy in Splitting by Site strategy. We cannot exclude the possibility that ComBat-like harmonization tools may overcorrect the data and remove weaker group differences of interest^38^. Hence, encouraging such evaluations in large data sets as well as implementing new methods to be tested^39,40^ on both the group and the single subject prediction level could be of great benefit for the imaging community.

### Machine learning performance

In our study, the selection of shallow linear and non-linear classification algorithms was guided its low computational complexity and robustness. According to previous studies^14,17^, SVM is the most commonly and successfully used algorithm in previous analyses. We have tested other commonly used linear ML algorithms, such as logistic regression with LASSO, logistic ridge regression and elastic net logistic^14,41,42^. Given that logistic regression models already have an in-built feature selection procedure, we also included feature selection algorithms such as the two-sample t-test and PCA^43–45^, for a fair comparison with SVM. Lastly, we included kernel SVM and random forest as representative shallow non-linear models. *There was no single winner with a significantly higher classification performance across all algorithms*, with a balanced accuracy up to 64%, when applied in data split by age/sex, and up to 53%, when split according to subsets of site. A similar trend was observed with AUC. In general, specificity was up to 5% higher than sensitivity, possibly because of the imbalanced MDD/HC data sets, even when the impact of both classes was weighted by its ratio during the training.

Considering such a low balanced accuracy, future studies could apply more sophisticated classification methods such as Convolutional Neural Networks^46^, which are able to detect nonlinear interactions between all the features as well as to consider spatial information of the given features. As it was demonstrated previously on both real and simulated data^47^, regressing out covariates can lead to lower classification performance, therefore one could use an importance weighting instead. Another option would be to include other data modalities such as vertex-wise cortical and subcortical maps^48,49^ or even voxel-wise T1 images to capture even more fine-grained changes^50^, which are also present in shapes of subcortical structures^51^ or diffusion MRI^52^. A recent resting-state fMRI multi-site study by Qin^53^ reported an accuracy of 81.5%*.* Thus, integration of structural and functional data modalities may result in even higher classification performances.

### Predictive brain regions

Our results do not support the hypothesis that MDD can be discriminated from HC by regional structural features; classification performance, when site effects were removed, was close to chance level. Nevertheless, during investigation of the most discriminative regions, even after ComBat, we found an overlap with previously reported MDD-related regions. Multiple cortical and subcortical regions were found as the most discriminative between MDD and HC. Most of the cortical regions were identified in previous ENIGMA MDD work^10^, which overlaps with our study set of cohorts. Shape differences in left temporal gyrus were reported previously in a younger population with MDD^54^. Left postcentral gyrus and right cuneus surface area were associated with severity of depressive symptoms, while left superior frontal gyrus, bilateral lingual gyrus and left entorhinal cortical thickness were decreased in MDD group^10,55^. In a previous study, MDD subjects exhibited reduced cortical volume compared to HC^56^. Differences in orbitofrontal cortex between MDD and HC were also previously identified^10^. Overall, the effect sizes for case–control differences in these studies were small, which is in line with our current results showing low classification accuracies of these structural brain measures. Additionally, we also found increased thickness of left caudal middle frontal gyrus, right pars triangularis, right superior parietal and right temporal pole in MDD group, which was not previously reported. All subcortical volumes identified as informative for classification became uninformative after ComBat was applied, suggesting that either previously identified alterations in subcortical regions^11^ cannot be directly used as MDD predictors at an individual level or ComBat removed differences significant for classification. One possible reason is that subcortical volumes tend to exhibit complex association with the age. Therefore, linear age regression might be an overly simplistic representation of aging trajectories both in ComBat and residualization step. While some of the regions were found also to be predictive in the previous large sample MDD versus HC study from Stolicyn and colleagues^17^, it is dificult to draw a consistent conclusion as they highlight the regions based on the selection frequency by the decision tree model, without reporting the direction of the modulation.

When models were trained and tested only on the subset of features in Splitting by Age/Sex, cortical thicknesses and subcortical volumes yielded higher balanced accuracy compared to cortical surface areas, which is consistent with the previous Enigma MDD meta-analysis, due to an overlap of study cohorts. When data was harmonized, there was no distinct subgroup of features providing more discriminative information. Together, we observed more changes in weights for cortical thicknesses and subcortical volumes after applying ComBat. One possibility is that differences are more pronounced in thickness than surface area, which is in line with previous findings from univariate approaches^57^. Another possibility is that differences in scanners and acquisition protocols may affect thickness features more strongly than surface areas, in line with previous works^58^. This is a very pertinent topic to be further investigated using multi-cohort mega-analyses on volumetric measures, particularly when the site effect is systematically considered.

Importantly, identified features correspond to the Splitting by Age/Sex strategy as the SVM model was trained on the whole data set with entirely mixed cohorts. While these regions were found to be informative according to the SVM with linear kernel, this model (and every other considered model) failed to differentiate MDD from HC on an individual level, thus one has to be cautious when interpreting these results. When we trained the SVM model with a linear kernel using data exclusively from a single site, a strong correspondence was not evident among the weights derived from various sites. This lack of sustained differences in individual weights underscores the absence of pronounced structural alterations even when the models are trained on more homogenous sets. Structural alterations in myelination, gray matter, and curvature were found to be associated with MDD-associated genes^59^. Furthermore, a small sample study revealed MDD-related alterations in sulcal depth^60^, while white matter topologically-based MDD classification led to up to 76% in accuracy^61^. Thus, the performance could be potentially elevated by integrating morphological shape features with white matter characterestics, such as sulcal depth and curvature, and myelination density as it led to improved performance when classifying sex and autism^62^.

### Data stratification

When the data set was stratified, we found substantial differences in balanced accuracies between the groups only for Splitting by Age/Sex strategy without harmonization step, yet these results were strongly influenced by the site effect. Harmonization step equalizes the accuracies within all pairs of comparisons to a roughly chance probability. Same balanced accuracy was observed when the Splitting by Site strategy was used. This suggests that the demographic and clinical subgroups that we considered do not contain information to predict MDD on an individual level and do not differ in terms of the resultant accuracy, at least according to simplest ML models, despite the group level differences reported prior^10,63^. Large sample meta-analysis of white mater characteristics that investigated similar subgroups also did not reveal significant differences^64^, suggesting that the inclusion of these features into ML analysis might not be beneficial for classification improvement. Similarly, a large sample MDD classification study including structural and functional neuroimaging data did not reveal any significant differences between males and females^65^. However, we speculate that other clinically relevant stratifications such as the number of depressive episodes^53,66^ and course of disease^53,67^ using functional data in further large studies may improve classifications.

## Conclusion

We benchmarked the classification of MDD versus HC using shallow linear and non-linear ML models applied to regional surface area features, cortical thickness features and subcortical volumes in the largest multi-site global data set to date. We systematically addressed the questions of A. general MDD characteristics and B. generalizability of models to perform on unseen sites by splitting the data according to A. demographic information (Splitting by Age/Sex) and B. site affiliation (Splitting by Site), which were complemented by ComBat harmonization. A classification accuracy up to 63% was achieved when all cohorts were present in the test set, which decreased down to 52% after ComBat harmonization. Here we have shown that most commonly used ML algorithms may not be able to differentiate MDD from HC on the single subject level using only structural morphometric brain data, even when trained on data from thousands of participants. Furthermore, the performance was not higher in stratified, clinically and demographically more homogeneous groups. Additional work is required to examine if more sophisticated algorithms also known as deep learning can achieve higher predictive power or if other MRI modalities such as task-based or resting state fMRI can provide more discriminative information for successful MDD classification.

## Material and methods

### Participant sample

A total of 5365 participants, 2288 patients with MDD and 3077 healthy controls, from 30 cohorts participating in the ENIGMA MDD working group, were included in the analyses. Information on sample characteristics, inclusion/exclusion criteria for each cohort can be found in Supplementary Table 1. Subjects with less than 75% of combined cortical and subcortical features and/or missing demographic/clinical information required for a particular analysis were excluded from the analysis. We implemented 75% as a reasonable cut-off value, which allowed us to accommodate a large amount of the available data without incurring biased model estimations. Furthermore, after exclusion of the subjects with less than 75% of existing data, total number of missing values was less than 10% from the remaining participants. According to the third guideline by Newman^68^, (i.e., "for construct-level missingness that exceeds 10% of the sample, ML and multiple imputation (MI) techniques should be used under a strategy that includes auxiliary variables and any hypothesized interaction terms as part of the imputation/estimation model"), we performed data imputation by considering age and sex factors as "auxiliary variables". Missing cortical and subcortical features for the remaining subjects (2% of all data) were imputed by using multiple linear regression with age and sex of all subjects (regardless of diagnosis) as predictors, estimated for each cohort separately. The Ethics Committee of the University Medical Center (UMG), Germany, approved the study. In accordance with the Declaration of Helsinki, all participating cohorts confirmed approval from their corresponding institutional review boards and local ethics committees as well as collected written consent of all participants. In case of participants under 18 years old, a parent and/or legal guardian also gave the written consent.

### Brain imaging processing

Structural T1-weighted 3D brain MRI scans of participating subjects were acquired from each site and preprocessed according to the rigorously validated ENIGMA Consortium protocols (http://enigma.ini.usc.edu/protocols/imaging-protocols/). Information on the MRI scanners and acquisition protocols used for each cohort can be found in Supplementary Table 2. To facilitate the ability to pool the data from different cohorts, cortical and subcortical parcellation was performed on every subject via the freely available FreeSurfer (Version 5.1,5.3, 6 and 7.2) software^69,70^. Every cortical and subcortical brain parcellation was visually inspected as part of a careful quality check (QC) and statistically evaluated for outliers, according to the ENIGMA Consortium protocol (https://enigma.ini.usc.edu/protocols/imaging-protocols/). Cortical gray matter segmentation was based on the Desikan–Killiany atlas^71^, yielding cortical surface area and cortical thickness measures for 68 brain regions (34 for each hemisphere), resulting in 136 cortical features. Subcortical segmentation was based on the *Aseg* atlas^71^, providing volumes of 40 regions (20 for each hemisphere), of which we included 16: lateral ventricle, thalamus, caudate, putamen, pallidum, hippocampus, amygdala, and nucleus accumbens, bilaterally.

### Data splitting into cross-validation folds

We applied two different strategies to split the data into training and test sets: *Splitting by Age/Sex* and *Splitting by Site*. For both strategies, data was split into 10 folds, 9 of which were used for the training and the remaining fold was used as a test set. This was repeated iteratively until each fold was used once as a test set, thus performing the tenfold CV. We investigated the general differences in brain volumes that can characterize MDD by using the Splitting by Age/Sex strategy. In this way, the age and sex distribution as well as number of subjects between the folds were balanced to mitigate the effect of these factors on the classification performance. However, it should be noted that with each site represented in every CV fold the potential site effects in this strategy, if any, would be diluted between the folds, which would not represent a realistic clinical scenario, where a classification model likely has to generalize to unseen sites. Therefore, we used a second strategy, Splitting by Site, which would yield more realistic metrics of classification performance for unseen sites. Using this strategy, every site was present only in one fold, thus the model is always trained and tested on different sets of sites and sites were distributed across folds to balance the number of subjects in every fold as close as possible. In this scenario, potential site-specific confounders (e.g., different MR scanners/acquisition protocols, demographic and clinical differences, etc.) were not equally distributed between the training and test sets. In this way, we can fairly evaluate the generalizability from one cohort to another. Finally, to assess the performance estimates for each site, we explored leave-site-out CVs. Further details on both splitting strategies can be found in Supplementary Section “CV splitting strategies”.

### Classification models

We have chosen representative examples of shallow linear and non-linear classification models to establish a benchmark of MDD versus HC classification. For the linear models, we selected SVM with linear kernel^72^, and logistic regression with different types of regularization: L1 (LASSO), L2 (Ridge), and L1 + L2 (Elastic Net)^73^. Both SVM and LASSO models are commonly used classification models used in neuroimaging^14^ due to their low computational complexity. As regularization serves as an in-built feature selection algorithm, we evaluated SVM with additional feature selection via PCA and t-test. As many classification tasks are not linearly separable, potentially including MDD versus HC, we additionally evaluated robust shallow non-linear models, including SVM with RBF kernel^74^, and ensemble classification algorithm—random forest^75,76^. While, other shallow linear/non-linear models were evaluated for MDD versus HC classification task previously^14^, including linear discriminant analysis (LDA)^77^, SVM with other non-linear kernels, a large sample benchmark analysis revealed no significant advantage of their application in the general neuroimaging setting^78^.

### Analysis pipeline

**Figure 3:**
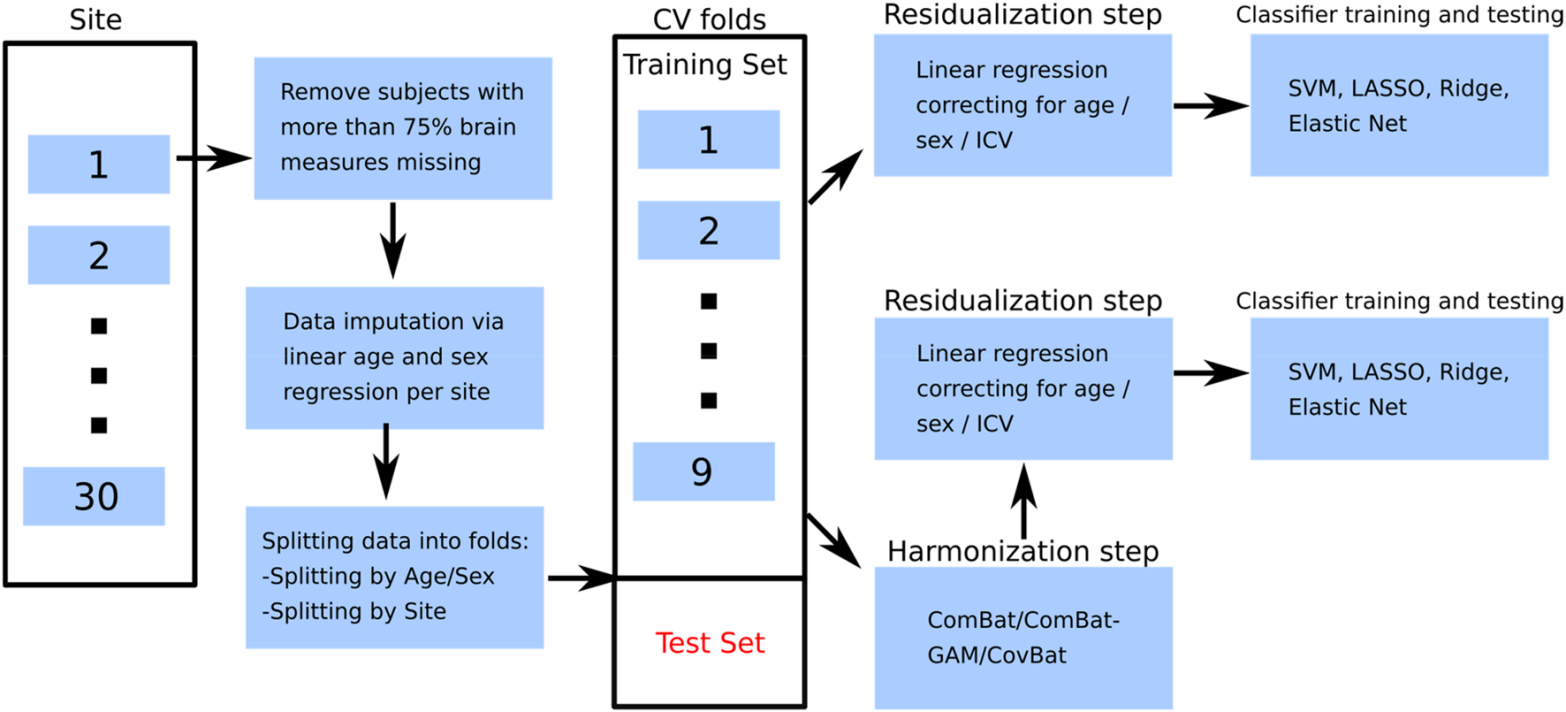
Detailed analysis pipeline. Initial data from all cohorts is split into training and test sets according to splitting strategies (Splitting by Age/Sex and Splitting by Site) after removing subjects with more than 75% missing data and data imputation steps. The corresponding training folds are then residualized directly to remove ICV, age and sex related effects and fed to the classification algorithms. In case of harmonization by ComBat, the residualization step takes place after the harmonization step is conducted. If training folds were harmonized by ComBat, the test fold was harmonized as well by using ComBat estimates from the training folds. Next, the test fold was residualized by using estimates obtained from the training folds. We estimated classification performance on the residualized test fold. This routine was performed iteratively for each combination of training and test folds.

After distributing the data into CV folds corresponding to the splitting strategies, 9 folds were used for the training, while the remaining fold was held out as a test set (Fig. 3). CV folds were residualized normatively, partialling out the linear effect of age, sex and ICV from all cortical and subcortical features. In this step, age, sex and ICV regressors were estimated on the HC from training CV folds and applied to remove the effect of age, sex, and ICV from brain measures in the MDD training data and all test data. After normalizing all features to have mean of zero and standard deviation of one based on the mean and standard deviation estimates from the training set initial features’ distributions, training and test folds were used for training and performance estimation, respectively. Additionally, class weighting was performed to mitigate an unbalanced training set across classes. Models’ hyperparameters were estimated in the training data via nested 10-folds cross-validation using grid search (random splits, for both Splitting by Site and Splitting by Age/Sex), before the performance was measured on the test data to avoid data leakage through the choice of hyperparameters. The list of hyperparameters that were adjusted can be found in Supplementary Table 3. We evaluated the performance of SVM with linear kernel, SVM with rbf kernel, logistic regression with LASSO regularization, logistic regression with ridge regularization, elastic net, and random forest by using balanced accuracy, sensitivity, specificity and AUC as performance metrics. For the model-level assessment^79^, all models were also trained on the subset of features, i.e. only cortical surface areas, only cortical thicknesses and only subcortical volumes. Lastly, we investigated which features contributed most to the classification performance by looking at the decision-making of the most successful model, in line with established guidelines^79^. In case no performance differences across models were found, we reported the weights of the SVM with linear kernel as the representative classifier. These weights correspond to the classification performance of Splitting by Age/Sex strategy as all sites are used for weight’s estimation. To assess confidence intervals of the feature weights, we performed 599-bootstrap^80,81^ on the whole data set.

Further analyses were performed by stratifying the data according to demographic and clinical categories, including sex, age of onset (< 21 years old vs > 21 years old), antidepressant use (yes/no at time of scan), and number of depressive episodes (first episode vs recurrent episodes). The subjects with missing information on these factors were not included in these analyses, while they were still considered for the main analysis.

All the steps from CV folds to classification were repeated with feature specific harmonization of site effects via ComBat. Variance explained by age, sex and ICV was preserved in the cortical and subcortical features during harmonization step. The harmonized folds were then residualized normatively with all subsequent steps from the analysis without harmonization step. Furthermore, we compared ComBat with two modifications: ComBat-GAM and CovBat. More detailed description of ComBat, ComBat-GAM and CovBat as well as their implementation for both splitting strategies can be found in Supplementary section “Harmonization methods”.

We used Python (version 3.8.8) to perform all calculations. All classification models and feature selection methods were imported from sklearn library (version 1.1.2). We modified ComBat script (https://github.com/Jfortin1/ComBatHarmonization) to incorporate ComBat-GAM (https://github.com/rpomponio/neuroHarmonize) and CovBat (https://github.com/andy1764/CovBat_Harmonization) in one function for both splitting strategies.

### Supplementary Information

Supplementary Information.

## Data Availability

Organizers of the ENIGMA-MDD (https://enigma.ini.usc.edu/ongoing/enigma-mdd-working-group/) should be contacted directly to request data from this study. Authors are not authorized to share data from participating sites with third parties inside or outside the ENIGMA-MDD consortium.
