# Supplementary material for: Multi-site benchmark classification of major depressive disorder using machine learning on cortical and subcortical measures
Source: Sci Rep. 2024 Jan 11;14:1084. doi: 10.1038/s41598-023-47934-8 (PMC10784593; doi:10.1038/s41598-023-47934-8)
Supplement: Supplementary file 1 — Supplementary Information. [file 41598_2023_47934_MOESM1_ESM.docx]

**Supplementary Materials**

**Multi-site benchmark classification of major depressive disorder using machine learning on cortical and subcortical measures**

Vladimir Belov^1^, Tracy Erwin-Grabner^1^, Moji Aghajani^2,3^,Andre Aleman^4^, Alyssa R. Amod^5^, Zeynep Basgoze^6^, Francesco Benedetti^7^, Bianca Besteher^8^, Robin Bülow^9^, Christopher R. K. Ching^10^,Colm G. Connolly^11^, Kathryn Cullen^6^, Christopher G. Davey^12^, Danai Dima^13,14^, Annemiek Dols^2^, Jennifer W. Evans^15^, Cynthia H. Y. Fu^16,17^, Ali Saffet Gonul^18^, Ian H. Gotlib^19^, Hans J. Grabe^20^, Nynke Groenewold^5^, J. Paul Hamilton^21,22^, Ben J. Harrison^12^, Tiffany C. Ho^23,24^, Benson Mwangi^25,26^, Natalia Jaworska^27^, Neda Jahanshad^10^, Bonnie Klimes-Dougan^28^, Sheri-Michelle Koopowitz^5^, Thomas Lancaster^29,30^, Meng Li^8^, David E. J. Linden^29,30,31,32^, Frank P. MacMaster^33^, David M. A. Mehler^29,30,34^, Elisa Melloni^7^, Bryon A. Mueller^6^, Amar Ojha^35,36^, Mardien L. Oudega^2^, Brenda W. J. H. Penninx^2^, Sara Poletti^7^, Edith Pomarol-Clotet^37^, Maria J. Portella^38^, Elena Pozzi^39,40^, Liesbeth Reneman^41^, Matthew D. Sacchet^42^, Philipp G. Sämann^43^, Anouk Schrantee^41^, Kang Sim^44,45,46^, Jair C. Soares^26^, Dan J. Stein^47^, Sophia I. Thomopoulos^10^, Aslihan Uyar-Demir^18^, Nic J. A. van der Wee^48^, Steven J. A. van der Werff^48,49^, Henry Völzke^50^, Sarah Whittle^51^, Katharina Wittfeld^20,52^, Margaret J. Wright^53,54^, Mon-Ju Wu^25,26^, Tony T. Yang^23^, Carlos Zarate^55^, Dick J. Veltman^2^, Lianne Schmaal^39,40^, Paul M. Thompson^10^, and Roberto Goya-Maldonado^1,*^, for the ENIGMA Major Depressive Disorder working group^56^

Affiliations:

^1^ Laboratory of Systems Neuroscience and Imaging in Psychiatry (SNIP-Lab), Department of Psychiatry and Psychotherapy, University Medical Center Göttingen (UMG), Georg-August University, Von-Siebold-Str. 5, 37075 Göttingen, Germany;

^2^ Department of Psychiatry, Amsterdam UMC, Vrije Universiteit Amsterdam, Amsterdam Neuroscience, Amsterdam Public Health Research Institute, Amsterdam, The Netherlands;

^3^ Institute of Education and Child Studies, Section Forensic Family and Youth Care, Leiden University, Leiden, The Netherlands;

^4^ Department of Biomedical Sciences of Cells and Systems, University Medical Center Groningen, University of Groningen, Groningen, The Netherlands;

^5^ Department of Psychiatry and Mental Health, University of Cape Town, Cape Town, South Africa;

^6^ Department of Psychiatry and Behavioral Science, University of Minnesota Medical School, Minneapolis, MN, USA;

^7^ Division of Neuroscience, IRCCS San Raffaele Scientific Institute, Milano, Italy;

^8^ Department of Psychiatry and Psychotherapy, Jena University Hospital, Jena, Germany;

^9^ Institute for Radiology and Neuroradiology, University Medicine Greifswald, Greifswald, Germany;

^10^ Imaging Genetics Center, Mark and Mary Stevens Neuroimaging and Informatics Institute, Keck School of Medicine, University of Southern California, Marina del Rey, CA, USA;

^11^ Department of Biomedical Sciences, Florida State University, Tallahassee, FL, USA;

^12^ Melbourne Neuropsychiatry Centre, Department of Psychiatry, The University of Melbourne, Parkville, VIC, Australia;

^13^ Department of Psychology, School of Arts and Social Sciences, City, University of London, London, UK;

^14^ Department of Neuroimaging, Institute of Psychiatry, Psychology and Neuroscience, King’s College London, London, UK;

^15^ Experimental Therapeutics and Pathophysiology Branch, National Institute for Mental Health, National Institutes of Health, Bethesda, MD, USA;

^16^ School of Psychology, University of East London, London, UK;

^17^ Centre for Affective Disorders, Institute of Psychiatry, Psychology and Neuroscience, King’s College London, London, UK;

^18^ SoCAT Lab, Department of Psychiatry, School of Medicine, Ege University, Izmir, Turkey;

^19^ Department of Psychology, Stanford University, Stanford, CA, USA;

^20^ Department of Psychiatry and Psychotherapy, University Medicine Greifswald, Greifswald, Germany;

^21^ Center for Social and Affective Neuroscience, Department of Biomedical and Clinical Sciences, Linköping University, Linköping, Sweden;

^22^ Center for Medical Imaging and Visualization, Linköping University, Linköping, Sweden;

^23^ Department of Psychiatry and Behavioral Sciences, Division of Child and Adolescent Psychiatry, Weill Institute for Neurosciences, University of California, San Francisco, San Francisco, CA, USA;

^24^ Department of Psychology, University of California, Los Angeles, CA, USA;

^25^ Louis A. Faillace, MD, Department of Psychiatry and Behavioral Sciences, The University of Texas Health Science Center at Houston, Houston, TX, USA;

^26^ Center Of Excellence On Mood Disorders, Louis A. Faillace, MD, Department of Psychiatry and Behavioral Sciences at McGovern Medical School, The University of Texas Health Science Center at Houston, TX, USA;

^27^ Department of Psychiatry, McGill University, Montreal, Quebec, Canada;

^28^ Department of Psychology, University of Minnesota, Minneapolis, MN, USA;

^29^ Cardiff University Brain Research Imaging Centre, Cardiff University, Cardiff, UK;

^30^ MRC Center for Neuropsychiatric Genetics and Genomics, Cardiff University, Cardiff, UK;

^31^ Division of Psychological Medicine and Clinical Neurosciences, Cardiff University, Cardiff, UK;

^32^ School of Mental Health and Neuroscience, Faculty of Health, Medicine and Life Sciences, Maastricht University, Maastricht, The Netherlands;

^33^ Departments of Psychiatry and Pediatrics, University of Calgary, Calgary, AB, Canada;

^34^ Department of Psychiatry, Psychotherapy and Psychosomatics, Medical School, RWTH Aachen University, Aachen, Germany;

^35^ Center for Neuroscience, University of Pittsburgh, Pittsburgh, PA, USA;

^36^ Center for Neural Basis of Cognition, University of Pittsburgh, Pittsburgh, PA, USA;

^37^ FIDMAG Germanes Hospitalàries Research Foundation, Centro de Investigación Biomédica en Red de Salud Mental (CIBERSAM), Barcelona, Catalonia, Spain;

^38^ Sant Pau Mental Health Research Group, Institut de Recerca de l'Hospital de la Santa Creu i Sant Pau, Barcelona, Catalonia, Spain. CIBERSAM, Madrid, Spain;

^39^ Centre for Youth Mental Health, The University of Melbourne, Parkville, VIC, Australia;

^40^ Orygen, Parkville, VIC, Australia

^41^ Amsterdam University Medical Centers, location AMC, Department of Radiology and Nuclear Medicine, Amsterdam, the Netherlands;

^42^ Meditation Research Program, Department of Psychiatry, Massachusetts General Hospital, Harvard Medical School, Boston, MA, USA;

^43^ Max Planck Institute of Psychiatry, Munich, Germany;

^44^ West Region, Institute of Mental Health, Singapore;

^45^ Yong Loo Lin School of Medicine, National University of Singapore, Singapore;

^46^ Lee Kong Chian School of Medicine, Nanyang Technological University, Singapore;

^47^ SA MRC Research Unit on Risk and Resilience in Mental Disorders, Department of Psychiatry and Neuroscience Institute, University of Cape Town, Cape Town, South Africa;

^48^ Leiden Institute for Brain and Cognition, Leiden University Medical Center, The Netherlands;

^49^ Department of Psychiatry, Leiden University Medical Center, Leiden, Netherland;

^50^ Institute for Community Medicine, University Medicine Greifswald, Greifswald, Germany;

^51^ Melbourne Neuropsychiatry Centre, Department of Psychiatry, The University of Melbourne and Melbourne Health, Melbourne, VIC, Australia;

^52^ German Center for Neurodegenerative Diseases (DZNE), Site Rostock/ Greifswald, Greifswald, Germany;

^53^ Queensland Brain Institute, The University of Queensland, Brisbane, QLD, Australia;

^54^ Centre for Advanced Imaging, The University of Queensland, Brisbane, QLD, Australia;

^55^ Section on the Neurobiology and Treatment of Mood Disorders, National Institute of Mental Health, Bethesda, MD, USA;

^56^ https://enigma.ini.usc.edu/ongoing/enigma-mdd-working-group/

***Corresponding author:**

PD Dr. Roberto Goya-Maldonado

Laboratory of Systems Neuroscience and Imaging in Psychiatry (SNIP-Lab)

Department of Psychiatry and Psychotherapy

University Medical Center Göttingen (UMG)

Von-Siebold Str. 5, 37075 Göttingen

e-mail: [roberto.goya@med.uni-goettingen.de](mailto:roberto.goya@med.uni-goettingen.de)

Supplementary Table 1: ENIGMA MDD Instrument for diagnosing major depressive disorder and exclusion criteria by site

| **Cohort** | **Diagnosis measurement** | **Sample characteristics/Inclusion criteria** | **Exclusion criteria** |
| --- | --- | --- | --- |
| **AFFDIS** | ICD-10/DSM-IV criteria | MDD subjects currently depressed and in day program or inpatient | All subject’s exclusion criteria: current or history of neurological disorder or brain injury, current substance abuse or dependence (not including nicotine), pregnancy, MRI contraindications, inability to give consent. MDD specific: comorbid psychiatric diagnosis. Healthy control specific: current or history of psychiatric diagnosis. |
| **Pharmo (AMC)** | MINI Plus | 48 subjects with lifetime diagnosis of either MDD and/or AD and 14 healthy controls. Patients were startified depending on exposure to SSRIs: early (before age 23) or late (after age 23) exposure to SSRI's, or no exposure at all (UN). 15 subjects were diagnosed with only MDD, 3 with only AD and 22 with both MDD and AD (8 subjects did not receive a diagnosis due to incomplete M.I.N.I. Plus assessment). According to the M.I.N.I. Plus, none of the HC subjects were ever diagnosed with MDD or AD | Less than three-week medication-free interval before scanning, current psychotropic medication use, a history of chronic or neurological disorder, family history of sudden heart failure or epileptic attacks, pregnancy (tested via urine sampling prior to the assessment), breast feeding, alcohol dependence and contra-indications for an MRI scan (e.g., ferromagnetic fragments). Participants agreed to abstain from smoking, caffeine and alcohol use for 24 hours prior to the assessments. |
| **Barcelona** | DSM-IV-TR acc. to CIDI-interview and HAMD | Outpatients with MDD diagnosis (DSM-IV-TR), with a first episode, recurrent MDD or chronic MDD (TRD) age 18-65 | The exclusion criteria for healthy participants were: lifetime psychiatric diagnoses, first-degree relatives with psychiatric diagnoses and clinically significant physical or neurological illnesses. Axis I comorbidity according to DSM-IV-TR criteria was an exclusion criterion for all participants. |
| **Cardiff** | Hamilton Depression Rating Scale (HDRS-17) | N= 40, MDD patients with a current moderate to severe depressive episode despite minimum three months of stable antidepressant treatment | Psychotic symptoms, current substance dependence, eating disorders, claustrophobia and other MRI contraindications, and ongoing non-pharmacological treatment. |
| **CSAN (Adf)** | MINI | Current MDD: Meets MINI criteria for depression; comorbid anxiety disorders are allowed; mood-congruent psychotic symptoms allowed. | Current MDD: a current DSM-5 diagnosis of substance use disorder, except nicotine; a psychotic disorder, except depression with mood-congruent psychotic features; new antidepressant medication during the month before study participation (two months for fluoxetine); change of the dose of psychotropic medications over the last month (antidepressant and antipsychotic medication) or the last two months (mood stabilizers and anticonvulsants). |
| **Calgary** | KSADS | First episode MDD and healthy controls (Dalhousie sample). Recurrent MDD and healthy controls, recruited via referral from clinicians in Calgary, Alberta and through advertisements in local clinics and at the University of Calgary (Calgary sample). | Dalhousie Sample: A history of neurological illness, medical illness, claustrophobia, >21 year of age, or the presence of a ferrous implant or pacemaker. University of Calgary: Left handed; history of seizures, epilepsy or other neurological or psychiatric diagnoses (specifically bipolar disorder, psychosis, pervasive developmental disorder, eating disorders, PTSD); pregnancy |
| **DCHS** | MINI | Women over the age of 18 years, who were between 20 and 28 weeks pregnant, who presented at either of the two recruitment clinics, and who had no intention of moving out of the area within the following year, and were able to give written consent | 1) loss of consciousness longer than 30 minutes, 2) inability to speak English, 3) current/lifetime alcohol and/or substance dependence or abuse, 4) psychopathology other than PTSD and/or MDD, 5) traumatic brain injury, 6) standard MRI exclusion criteria |
| **ETPB** | HAMD,BDI, SHAPS,MADRS | Treatment resistant depression, at least one failed trial MADRS >20 | Current or past diagnosis of Schizophrenia or any other psychotic disorder as defined in the DSM-IV. Subjects with a history of DSM-IV drug or alcohol dependency or abuse (except for nicotine or caffeine) within the preceding 3 months. Female subjects who are either pregnant or nursing. Serious, unstable illnesses including hepatic, renal, gastroenterological, respiratory, cardiovascular (including ischemic heart disease), endocrinological, neurologic, immunologic, or hematologic disease. Subjects with uncorrected hypothyroidism or hyperthyroidism. Subjects with one or more seizures without a clear and resolved etiology. Treatment with a reversible MAOI within 4 weeks prior to study phase I. Treatment with fluoxetine within 5 weeks prior to study phase I. Treatment with any other concomitant medication not allowed (Appendix A for Substudy 2; Appendix G for Substudy 4) 14 days prior to study phase I. No structured psychotherapy will be permitted during the study. Current NIMH employee/staff or their immediate family member. Additional Exclusion Criteria for substudy 2 (patients with MDD) Previous treatment with ketamine or hypersensitivity to amantadine. Additional Exclusion Criteria for Substudy 4 (patients with MDD or BD). Subjects who currently are using drugs (except for caffeine or nicotine), must not have used illicit substances in the 2 weeks prior to screen and must have a negative alcohol and drug urine test (except for prescribed benzodiazepines) urine test at screening. Presence of any medical illness likely to alter brain morphology and/or physiology (e.g., hypertension, diabetes) even if controlled by medications. Clinically significant abnormal laboratory tests. Presence of metallic (ferromagnetic) implants (e.g., heart pacemaker, aneurysm clip). Subjects who, in the investigator s judgment, pose a current serious suicidal or homicidal risk, or who have a MADRS item 10 score of >4. |
| **EPISCA (Leiden)** | ADIS | Inclusion criteria for the patient group were: having clinical depression  as assessed by categorical and dimensional measures of DSM-IV depressive  and anxiety disorders, no  current and prior use of antidepressants, and being referred for CBT at  an outpatient care unit. Inclusion criteria for the control group were:  no current or past DSM-IV classifications, no clinical scores on validated  mood and behavioral questionnaires, no history of traumatic experiences,  and no current psychotherapeutic and/or psychopharmacological intervention of any kind. | Primary DSM-IV clinical diagnosis of ADHD, ODD, CD, pervasive developmental disorders, post-traumatic stress disorder, Tourette's syndrome, obsessive–compulsive disorder, bipolar disorder, and psychotic disorders; current substance abuse; history of neurological disorders or severe head injury; age < 12 or > 21 years; pregnancy; left-handedness; IQ score < 80 as measured by the Wechsler Intelligence Scale for Children (WISC) (Wechsler, 1991) or Adults (Wechsler, 1997); and general MRI contra- indications. |
| **FIDMAG** | DSM-IV-TR criteria | MDD patients within a current depressive episode (HDRS >= 17, only 1 patient was in remission), right-handed, age 18-65 | Patients were excluded (i) if they were left-handed; (ii) if they were younger than 18 or older than 65 years; (iii) if they had a history of brain trauma or neurological disease; (iv) if they had shown alcohol/ substance abuse within 12 months prior to participation; and (v) if they had undergone electroconvulsive therapy in the previous 12 months. |
| **Groningen sample (DIP)** | MINI-SCAN | Outpatients with MDD diagnosis. Inclusion MDD: Outpatients treated in mental health care for depression, BDI-II>13 at screening, adults. | Exclusion MDD: Comorbid axis-I disorders other than anxiety disorders or past substance abuse, other psychotropic medication than stable use of SSRI/SNRI/TCA, established cardiovascular disease, active and concrete suicidal plans, inadequate language proficiency, cognitive impairments or neurological disease that interferes with task performance. Exclusion CTL: Same as MDD, lifetime history of MDD, BDI>8. |
| **Houston** | SCID interview | Outpatients | MDD subjects: age below 18; lifetime or current diagnosis of psychotic disorder, or bipolar I or II disorder; substance abuse/dependence in 6 months prior to study inclusion; current major medical problems. Control subjects: age below 18; current major medical problems; current psychiatric or neurologic disorder; history of psychiatric disorders in a first-degree relative; current major medical problems. Both groups: MRI contra-indications |
| **TiPs (Jena, Germany)** | SCID interview | Psychiatric inpatients and tinnitus patients with MDD or a disorder of the depressive spectrum (also adjustment disorders as pointed out in the data table); psychiatrically healthy controls were derived from community and tinnitus patients | MDD subjects: presence of axis-I disorders other than MDD or adjustment disorders. Control subjects: no Axis-I diagnosis, no medication use. Exclusion criteria for all subjects included history of neurological disease (e.g. tumor, head trauma, epilepsy) or untreated internal medical conditions, intellectual and/or developmental disability. Only German native speakers were allowed to participate. |
| **BRCDECC London** | SCAN interview | Community based or outpatients, none were inpatients. MDD subjects: Less than two depressive episodes of at least moderate severity. Did not meet DSM-IV diagnostic criteria for recurrent major depressive disorder. Control group participants were clinically interviewed to ensure they had never experienced depressive symptoms.  Exclusion criteria for all participants were for contraindications to MRI; other exclusion criteria were a diagnosis of neurological disorder, head injury leading to loss of consciousness or conditions known to affect brain structure or function (including alcohol or substance misuse), ascertained during clinical interview. Potential participants were also excluded if they or a first-degree relative had ever fulfilled criteria for mania, hypomania, schizophrenia or mood-incongruent psychosis. | Contraindications to MRI, diagnosis of neurological disorder, head injury leading to loss of consciousness or conditions known to affect brain structure or function (including alcohol or substance misuse), if they or a first-degree relative had ever fulfilled criteria for mania, hypomania, schizophrenia or mood-incongruent psychosis. |
| **MODECT** | MINI | Older adults, aged above 55, with severe depression admitted to be treated with ECT | Exclusion criteria were another major DSM-IV-TR diagnosis, such as schizophrenia, bipolar or schizoaffective disorder and a history of major neurological illness (including Parkinson’s disease, stroke and dementia). |
| **MPIP** | M-CIDI/SCAN interview | M. A. R. S. sample: both first and recurrent episodes; RUD sample: only recurrent episodes with some patients scanned in remission | 1. Munich Antidepressant Response Signature (MARS) study MDD subjects (clinical consensus diagnosis or M-CIDI (since 2008)): depressive syndromes secondary to any medical or neurological condition (e. g., intoxication, drug abuse, stroke), the presence of manic, hypomanic or mixed affective symptoms, lifetime diagnosis of alcohol dependence, illicit drug abuse or the presence of severe medical conditions (e.g., ischemic heart disease). Patients with bipolar depression were excluded for the current MR study. Control subjects: age > 65, MMSE<27, presence of severe somatic diseases or lifetime history of the following axis I disorders as assessed by the M-CIDI interview: alcohol dependence, drug abuse or dependence, possible psychotic disorder, mood disorder, anxiety disorder including OCD and PTSD, somatoform disorder, dissociative disorder NOS, and eating disorder 2. Recurrent unipolar depression (RUD) study: MDD subjects (SCAN interview): presence of manic episodes, mood incongruent psychotic symptoms, the presence of a lifetime diagnosis of intravenous drug abuse and depressive symptoms only secondary to alcohol or substance abuse or to medical illness or medication. Control subjects: presence of severe somatic diseases or life-time history of anxiety and affective disorders according to the Composite International Diagnostic-Screener (CIDI-S). All subjects: gross incidental MR findings such as territorial infarction, tumor, hydrocephalus, malformations and anatomical deviations (e.g. enlarged ventricles) that prevent appropriate image processing were additional exclusion criteria. 3. MR images of 9 additional controls acquired at the LMU, Munich, meeting equivalent criteria as the RUD control sample were included. |
| **Melbourne** | SCID interview | Youth depression sample: 15-25 years of age. Recruited as part of 2 large RCTs (incl. YoDA-C - Davey et al., 2014; Trials) and scanned prior to treatment randomization. 60 patients unmedicated (YoDA-C). | MDD subjects: lifetime or current SCID-I diagnosis of psychotic disorder, or bipolar I or II disorder. Control subjects: any SCID-I diagnosis or medication use. Both groups: Acute or unstable medical disorder; general MRI contraindications |
| **Minnesota** | Schedule for Affective Disorders and Schizophrenia for School-Age Children–Present and Lifetime Version and the Children’s Depression Rating Scale–Revised (CDRS-R). | Adolescents with MDD and HCs aged 12 to 19 years were recruited to participate through community postings and referrals from local mental health services. Adolescents with MDD were eligible if they had a primary diagnosis of MDD and had not received any psychotropic medication treatment for the past 2 months. Healthy adolescents were eligible if they had no current or past psychiatric diagnoses and were frequency matched to the MDD group on age and sex | Exclusion criteria for both groups included the presence of a neurologic or other chronic medical condition, mental retardation, pervasive developmental disorder, substance use disorder, bipolar disorder, or schizophrenia |
| **Moral Dilemma** | SCID interview | Youth depression sample: 15-25 years of age; recruited from outpatient service. Controls recruited from general community. | MDD subjects: lifetime or current SCID-I diagnosis of psychotic disorder, or bipolar I or II disorder; current antidepressant medication use. Control subjects: any SCID-I diagnosis or medication use. Both groups: Acute or unstable medical disorder; general MRI contraindications |
| **NESDA** | CIDI interview | DSM-4 based diagnosis of MDD (6 month recency), using CIDI interview. 93 (60%) MDD patients have a comorbid ANX diagnosis. Age range 18-65 | N/A |
| **QTIM** | CIDI interview | Retrospective questionnaire about depression episodes combined with an MRI study. The best described MDD episode is defined as the worst one (according to individuals). We have up to 5 supplementary episodes (briefly) described. Sample composed of twins and relatives. Population-based sample | MDD subjects: presence of axis-I disorders other than MDD and anxiety disorders Control subjects: antidepressant use, psychiatric disorders All subjects: relatedness between subjects, left handedness, history of neurological or other severe medical illness, head injury or current or past diagnosis of substance abuse, use of cognition affecting medication and general MRI contraindications |
| **San Francisco UCSF** | KSADS (semi-structured interview based on DSM) for MDD, DISC/DPS for HCL | Outpatient/community-based sample with DSM diagnosis, mostly antidepressant-naive and approximately 60% of MDD have comorbid anxiety disorders | Exclusion criteria for all participants included: 1) use of pharmacotherapeutics for treating psychiatric conditions within the past 6 months, 2) misuse of drugs within two months prior to MRI scanning; 3) two or more alcoholic drinks per week within the previous month (as assessed by the Customary Drinking and Drug Use Record; CDDR) (Brown et al, 1998); 4) a full scale IQ score of less than 75 (as assessed by the Wechsler Abbreviated Scale of Intelligence; WASI) (Wechsler, 1999); 5) contraindications for MRI including ferromagnetic implants and claustrophobia; 6) pregnancy or the possibility of pregnancy; 7) left-handedness; 8) prepubertal status (as assessed as Tanner stages of 1 or 2) (Tanner, 1962); 9) inability to understand and comply with procedures; 10) neurological disorder (including meningitis, migraine, or HIV); 11) head trauma; 12) learning disability; 13) serious health problems; and 14) complicated or premature birth (i.e., birth before 33 weeks of gestation). The MDD group was subject to the additional exclusion criterion of a primary psychiatric diagnosis other than MDD. The HCL group was subject to the additional exclusion criteria of: 1) history of mood or psychotic disorders in a first- or second-degree relative (as assessed by the Family Interview for Genetics; FIGS) (Maxwell, 1992); and 2) current or lifetime DSM-IV-TR Axis I psychiatric disorder. |
| **SHIP** | M-CIDI interview | Population based longitudinal cohort study | MDD subjects: presence of axis-I disorders other than MDD, anxiety disorders, conversion, somatization and eating disorder. Control subjects: no lifetime diagnosis of depression, no antidepressants, and severity index=0 All subjects: We removed subjects with medical conditions (e.g. a history of cerebral tumor, stroke, Parkinson’s diseases, multiple sclerosis, epilepsy, hydrocephalus, enlarged ventricles, pathological lesions) or due to technical reasons (e.g. severe movement artifacts or inhomogeneity of the magnetic field). |
| **SHIP-TREND** | M-CIDI interview | Population based longitudinal cohort study | MDD subjects: no special exclusion criteria Control subjects: no lifetime diagnosis of depression, no antidepressants, and severity index=0 All subjects: We removed subjects with due to medical conditions (e.g. a history of cerebral tumor, stroke, Parkinson’s diseases, multiple sclerosis, epilepsy, hydrocephalus, enlarged ventricles, pathological lesions) or due to technical reasons (e.g. severe movement artifacts or inhomogeneity of the magnetic field). |
| **San Raffaele Milano OSR** | SCID interview | adult MDD depressed inpatients | Other diagnoses on Axis I; pregnancy, history of epilepsy, major medical and neurological disorders; absence of a history of drug or alcohol dependency or abuse within the last six months. inflammation-related symptoms, including fever and infectious or inflammatory disease; uncontrolled systemic disease; uncontrolled metabolic disease or other significant uncontrolled somatic disorder known to affect mood; somatic medications known to affect mood or the immune system, such as corticosteroids, non-steroid anti-inflammatory drugs and statins. |
| **Singapore** | SCID interview | Inclusion: 1) DSM IV dx of MDD (Patients) 2) Age: 21-65 3) English speaking 4) Provision of informed written consent | Exclusion criteria 1) History of significant head injury 2) Neurological diseases such as epilepsy, cerebrovascular accident 3) Impaired thyroid function 4) Steroid use 5) DSM IV alcohol or substance use or dependence 6) Contraindications to MRI (e.g. pacemaker, orbital foreign body, recent surgery/procedure with metallic devices/implants deployed) using standard MRI Request Form from NNI 7) Pregnant women 8) Claustrophobia |
| **SoCAT** | SCID interview | Inclusion criteria: DSM IV dx for MDD patients Age: 18-65 right-handed currently depressed or remitted; Control subjects: any history of psychiatric disorder | Exclusion criteria 1) History of significant head injury 2) Neurological diseases such as epilepsy, cerebrovascular accident 3) Other diagnoses on Axis I disorders4) |
| **Stanford FAA** | SCID interview | Community-based DSM-diagnosed sample | MDD subjects: presence of axis-I disorders other than MDD, anxiety and eating disorders. Control subjects: control individuals did not meet diagnostic criteria for any current psychiatric. Both groups: alcohol / substance abuse or dependence within six months prior to MRI scanning, history of head trauma with loss of consciousness > 5 min, aneurysm, or any neurological or metabolic disorders that require ongoing medication or that may affect the central nervous system (including thyroid disease, diabetes, epilepsy or other seizures, or multiple sclerosis), MRI contraindications, or bad MRI data (e.g., extreme movement). |
| **Stanford T1w Aggregate** | SCID interview | Community-based DSM-diagnosed sample | MDD subjects: presence of axis-I disorders other than MDD, anxiety and eating disorders. Control subjects: control individuals did not meet diagnostic criteria for any current psychiatric. Both groups: alcohol / substance abuse or dependence within six months prior to MRI scanning, history of head trauma with loss of consciousness > 5 min, aneurysm, or any neurological or metabolic disorders that require ongoing medication or that may affect the central nervous system (including thyroid disease, diabetes, epilepsy or other seizures, or multiple sclerosis), MRI contraindications, or bad MRI data (e.g., extreme movement). |
| **TIGER** | KSADS | Community-based DSM-diagnosed sample | All subjects: Exclusion criteria were premenarchal status (for females), history of concussion within the past 6 weeks or history of any lifetime concussion with loss of consciousness, contraindications to MRI scanning (e.g. braces, metal implants, or claustrophobia), serious neurological or intellectual disorders that could interfere with the participant's ability to complete study components. MDD subjects: meeting lifetime or current DSM-IV criteria for any Bipolar Disorder, Psychosis, or Alcohol Dependence, or DSM-5 criteria for Moderate Substance Use Disorder with substance-specific threshold for withdrawal. CTL subjects: any current or past DSM-IV Axis I Disorder and first-degree relative with confirmed or suspected history of depression, mania, psychosis, or substance dependence. |

Supplementary Table 2: ENIGMA MDD Image acquisition and processing by cohort

| **Cohort** | **Scanner type** | **Sequence T1** | **FreeSurfer version** | **Slice orientation** | **Operating system** |
| --- | --- | --- | --- | --- | --- |
| **AFFDIS** | 3T Siemens Magnetom TrioTim | 3D T1 (176 slices; TR = 2250 ms; TE = 3.26 ms; FOV 256; voxel size 1X1X1mm) | 5,3 | Sagittal | Linux CentOS |
| **Pharmo (AMC)** | 3T Philips | T1 sequence details: 3D-TFE sequence TR= 9.7 ms, TE=4.6ms, matrix 192x192, voxel size = 0.875 x 0.875 x 1.2 mm; 120 slices. Axial plane. Philips 3T Ingenia 16 channel coil | 5,3 | Transverse | freesurfer-Linux-centos6_x86_64-stable-pub-v5.3.0 |
| **Barcelona** | 3T Philips Achieva | 3D MPRAGE images (Whole-brain T1-weighted); TR=6.7ms, TE=3.2ms; 170 slices, voxel size 0.89X0.89X1.2 mm. Image dimensions 288X288X170; field of view: 256X256X204; slice thickness: 1.2 mm; with a sagittal slice orientation, T1 contrast enhancement, flip angle: 8º, grey matter as a reference tissue, ACQ matrix MXP = 256X240 and turbo-field echo shots (TFE) = 218. | 6 | Sagittal | Scientific Linux 5 |
| **Cardiff** | A 3 Tesla whole body MRI system (General Electric, Milwaukee, USA) with an 8-channel head coil was used at the Cardiff University Brain Research Imaging Centre (CUBRIC). | High-resolution anatomical scan (Fast Spoiled Gradient-Recalled-Echo [FSPGR] sequence): 178 slices, TE=3 ms, TR=7.9 ms, voxel size=1.0×1.0×1.0 mm3, FA=15°, FOV=256×256 | 5,3 |  | freesurfer-Linux-centos6_x86_64-stable-pub-v5.3.0 |
| **CSAN (Adf)** | 3T Siemens MAGNETOM PRISMA | Whole-head t1-weighted MPRAGE (TR = 2300 ms, TE = 2.34 ms, FOV 250 × 250 mm, voxel size = 0.9 × 0.868 × 0.868 mm, flip angle = 8°) | 7.2 | Sagittal | Ubuntu |
| **Calgary** | 1.5T Siemens Magnetom Vision. 3T GE Discovery MR750 | 1.5T: A sagittal scout series was acquired to test image quality. 3D fast low angle shot (FLASH) sequence was used to acquire data from 124 1.5 mm-thick contiguous coronal slices through the entire brain (echo time = 5ms, repetition time = 25ms, acquisition matrix = 256 x 256 pixels, field of view = 24 cm and flip angle = 40°). 3T: Anatomical imaging acquisition parameters: axial acquisition, repetition time (TR), 2200 milliseconds (ms); echo time (TE), 3.04 ms; TI, 766, 780; flip angle, 13 degrees; 208 partitions; 256 × 256 matrix; and field of view, 256. | 5,3 | Dalhousie sample, coronal; Calgary sample, axial | MacOs Sierra |
| **DCHS** | 3T Siemens Skyra | 3D multi-echo MPRAGE, voxel size 1 mm x 1mm x 1.5mm, TR = 2530 ms, TE = 1.69 x 3.55 x 5.41 x 7.27ms, FOV: 256x256mm, flip angle = 7° | 5.3 | Sagittal | Linux-centos6_x86_64 |
| **ETPB** | 3T, GE HDx | Fast spoiled gradient recalled echo (FSPGR). Slice Thickness: 1. Repetition Time: 8.836. Echo Time: 3.496. Inversion Time: 450. Magnetic Field Strength: 3. Spacing Between Slices: 1. Echo Train Length: 1. Percent Sampling: 100. Percent Phase Field of View: 100. Pixel Bandwidth: 195.312. Reconstruction Diameter: 256. Acquisition Matrix: 0,256,256,0. In-plane Phase Encoding Direction: ROW. Flip Angle: 13 | 5,3 | Sagittal | Linux |
| **EPISCA (Leiden)** | 3T Philips Achieva | a sagittal 3-dimensional gradient-echo T1-weighted image was acquired (repetition time = 9.8 ms; echo time = 4.6 ms; flip angle = 8°; 140 sagittal slices; no slice gap; field of view =256 × 256 mm; 1.17 × 1.17 × 1.2 mm voxels; duration = 4:56 min) | 5,3 | Sagittal | Ubuntu 14.04.5 LTS (Linux 3.13.0-153-generic x86_64) |
| **FIDMAG** | 1.5T, GE Signa | 3D T1: matrix size = 512 × 512, 180 contiguous axial slices, voxel resolution = 0.47 × 0.47 × 1mm, no slice gap, TE = 3.93ms, TR = 2000ms and inversion time (TI) = 710ms, flip angle = 15 degrees | 6 | Axial | Linux-centos6_x86_64 |
| **Groningen sample (DIP)** | 3T Philips | 3D T1-weighted scan (170 slices; TR = 9ms; TE = 3.6ms; 256x231 matrix of 1×1×1 mm voxels) | 5,3 | Sagittal | SUSE Linux X86_64 |
| **Houston** | subjects in 20000s: 1.5 T Philips Medical Systems Gyroscan Intera; subjects in 30000s: 3T Siemens Allegra | Subjects in the 20000s: Fast field echo sequence- repetition time (TR) = 24 ms, echo time (TE) = 4.99 ms, flip angle = 40°, slice thickness = 1 mm, matrix size = 256 × 256 and 150 slices. Subjects in 30000s: MPRAGE- repetition time (TR) = 1750 ms, echo time (TE) = 4.39 ms, flip angle = 8°, slice thickness = 1 mm, matrix size = 208 × 256 and 160 slices. | 5,3 | Subjects in 20000s: Sagittal; Subjects in 30000s: Transverse | Fedora 19 |
| **TiPs (Jena, Germany)** | 3T Siemens MAGNETOM Prisma_fit | MPRAGE sequence: TR 2300 ms, TE 3.03 ms, α 9°, 192 contiguous sagittal slices, in-plane field of view 256 mm, voxel resolution 1Å~1Å~1 mm; acquisition time 5:21 min | 5,3 | Sagittal | Linux |
| **BRCDECC London** | 1.5T GE Signa HDx | ADNI-1 MPRAGE pulse sequence (details at http://adni.loni.ucla.edu/research/protocols/mri-protocols/) | 5,3 | Sagittal | Linux-centos4_x86_64 |
| **MODECT** | 3T (General Electric Signa HDxt, Milwaukee, WI, USA) | T1-weigthed data set was acquired (flip angle=12°, repetition time=7.84 milliseconds, echo time=3.02 milliseconds; matrix 256x256, voxel size 0.94x0.94x1 mm; 180 slices). | 5,3 | Coronal | Linux |
| **MPIP** | 1.5T GE and Siemens (the latter: only few cases) | #1: T1-weighted SPGR sagittal 3D volume. TR=1030 msec; TE=3.4 msec; 124 slices; matrix=256x256; FOV=23.0x23.0 cm2; voxel size=0.8975 mm x0.8975 mm x 1.2- 1.4 mm; flip angle=90°; birdcage resonator. #2: same scanner as #1, platform update Signa Excite, sagittal T1-weighted (spin echo sequence, TR=9.7 msec, TE=2.1 msec; FOV=25.0x25.0 cm2, voxel size=0.875 mm x0.875 mm x1.2 mm, 124- 132 slices, flip angle=90°. #3: Siemens 1.5 Tesla, Vario, 3D MPRAGE, TR=11.6 msec; TE=4.9 msec; FOV 23x23 cm2; matrix 512x512; 126 axial slices; voxel site 0.45 mm x 0.45 mm x 1.5 mm. (only N=2 subjects) | 5,3 | 1.5 GE: sagittal. 1.5 Siemens: axial | Linux 2.6.37.1-1.2- desktop x86_64 |
| **Melbourne** | 3T GE Signa Excite | 3D BRAVO sequence 140; TR=7900 ms; TE=3000 ms; flip angle=13º; FOV=256 mm; matrix=256 x 256 | 5,3 | Axial | Linux Debian x86 64 |
| **Minnesota** | 3.0 Tesla Tim Trio scanner; Siemens Corp | A 5-minute structural scan was acquired using a T1-weighted, high-resolution, magnetization-prepared gradient-echo sequence: repetition time, 2530 milliseconds; echo time, 3.65 milliseconds; inversion time, 1100 milliseconds; flip angle, 7°; field of view, 256 × 176 mm; voxel size, 1-mm isotropic; 224 slices; and generalized, autocalibrating, partially parallel acquisition acceleration factor, 2. | 5,3 | Coronal | Linux |
| **Moral Dilemma** | 3T GE Signa Excite | 3D BRAVO sequence: 140 contiguous slices; repetition time, 7900 ms; echo time, 3000 ms; flip angle, 13°; in a 25.6-cm field of view, with a 256 × 256 pixel matrix and a slice thickness of 1 mm (1 mm gap). | 5,3 | Axial | Linux Debian x86 64 |
| **NESDA** | 3T Phillips Achieva/Intera | 3D gradient-echo T1-weighted sequence. TR=9 msec; TE=3.5 msec; flip angle 8º, FOV = 256 mm; matrix: 25x62x56; in plane voxel size = 1 mm × 1 mm x 1 mm; 170 slices. | 5 | Sagittal | SHARK HPC, Linux environment |
| **QTIM** | Bruker 4T Wholebody MRI | 3D T1 weighted sequence. TR=1500 msec; TE=3.35 msec; flip angle=8°, 256 or 240 (coronal or sagittal) slices, FOV=240 mm, matrix 256x256x256 (or 256x256x240) | 5,1 | Coronal, then sagittal following software upgrade. | Linux- centos4_x86_64- stable-pub-v5.1.0 |
| **San Francisco UCSF** | 3T GE Discovery MR750 | SPGR T1-weighted: TR=8.1 ms; TE=3.17 ms; TI=450 ms; flip angle=12°; 256x256 matrix; FOV=250x250 mm; 168 sagittal slices; slice thickness=1 mm; in-plane resolution=0.98x 0.98 mm | 5,3 | Sagittal | Linux-centos6_x86_64-stable-pub-v5.3.0. |
| **SHIP** | 1.5T Siemens Avanto | 3D T1-weighted (MP-RAGE/ axial plane); TR=1900 msec; TE=3.4 msec; Flip angle=15°; voxel size 1 mm x 1 mm x 1 mm | 5.3 (cortical), 5.1 (subcortical) | Axial | Centos6_x86_64 |
| **SHIP-TREND** | 1.5T Siemens Avanto | 3D T1-weighted (MP-RAGE/ axial plane); TR=1900 msec; TE=3.4 msec; Flip angle=15°; voxel size 1 mm x 1 mm x 1 mm | 5.3 (cortical), 5.1 (subcortical) | Axial | Centos6_x86_64 |
| **San Raffaele Milano OSR** | 3T Philips Ingenia and 3T Philips Intera scanner | 3D-MPRAGE sequence: TR 2500 ms, TE 4.6 ms, field of view FOV = 230 mm, matrix = 256 × 256, in-plane resolution 0.9 × 0.9 mm, yielding 220 transversal slices with a thickness of 0.8 mm. | 5,3 | axial | Linux Ubuntu 16.04 |
| **Singapore** | Achieva 3T, Philips Medical Systems, Netherlands | Whole brain high resolution 3D MP-RAGE (magnetisation-prepared rapid acquisition with a gradient echo) volumetric scans (TR/TE/TI/flip angle 8.4/3.8/3000/8; matrix 256x204; FOV 240mm2) with axial orientation (reformatted to coronal) | 5,3 | Axial | Linux_Ubuntu12.04_6 4 |
| **SoCAT** | 3.0 T, Siemens Verio,Numaris/4,Syngo MR B17,Erlangen,Germany | 3D T1 weighted MP-Rage/axial plane; TR=1900 msec; TE=3.4 msec; Flip angle=15°; Voxel size 1 mm x 1 mm x 1 mm | 5,3 | Axial | Ubuntu 18.04 LTS |
| **Stanford FAA** | 3.0T GE Discovery MR750 | Whole-brain T1-weighted images were collected using a spoiled gradient echo (SPGR) pulse sequence (186 sagittal slices; resolution = 0.9 mm isotropic; flip angle = 12°; repetition time [TR] = 6,240 ms; echo time [TE] = 2.34 ms) | 5,3 | Sagittal | Linux-centos6_x86_64 |
| **Stanford T1w Aggregate** | 1.5T GE Signa Excite | Whole-brain T1-weighted images were collected using a spoiled gradient echo (SPGR) pulse sequence (116 sagittal slices; through-plane resolution = 1.5 mm; in-plane resolution = 0.86 x 0.86 mm; flip angle = 15 degrees; repetition time [TR] = 8.3-10.1 ms; echo time [TE] = 1.7-3.0; inversion time [TI] = 300 ms; matrix = 256 x 192). | 5,3 | Sagittal | Centos6_x86_64, Linux-based HPC |
| **TIGER** | 3T GE MR750 | TR/TE/TI=8.2/3.2/600 ms; flip angle=12°; 156 axial slices; FOV=25.6 cm; matrix=256 mm x 256 mm, isotropic voxel=1 mm, total scan time: 3:40 | 6 | Axial | Linux |

Supplementary Table 3: List of hyperparameters of trained algorithms. Optimal hyperparameters were found by the grid search. We followed a heuristic approach outlined in ^1^ to determine a range of values for C and $\gamma$. To access different power of regularization, we followed the same range of values for $\lambda$. The range of values of random forest hyperparameters were optimized according to ^2^.

| **Classification algorithm** | **Feature Selection** | **Hyperparameters** | **Nested CV** |
| --- | --- | --- | --- |
| **SVM Linear** | None | C = [${10}^{-4},{10}^{-3}, \ldots, {10}^{4}$] | 10 fold |
| **SVM Linear** | PCA | C = [${10}^{-4},{10}^{-3}, \ldots, {10}^{4}$]  % components = [10%,20%, ... ,100%] | 10 fold |
| **SVM Linear** | Ttest (pvalue<0.05) | C = [${10}^{-4},{10}^{-3}, \ldots, {10}^{4}$] | 10 fold |
| **SVM rbf** | None | C = [${10}^{-4},{10}^{-3}, \ldots, {10}^{4}$]  $\gamma$= [${10}^{-4},{10}^{-3}, \ldots, 10$] | 10 fold |
| **LASSO** | None | $\lambda$ = [${10}^{-4},{10}^{-3}, \ldots, {10}^{4}$] | 10 fold |
| **Ridge** | None | $\lambda$ = [${10}^{-4},{10}^{-3}, \ldots, {10}^{4}$] | 10 fold |
| **Elastic Net** | None | $\lambda$ = [${10}^{-4},{10}^{-3}, \ldots, {10}^{4}$]  $\alpha$ = [$0.1, 0.2, \ldots, 1$] | 10 fold |
| **Random Forest** | None | max depth = [40,50,...,100]  number of trees = [400,600,..., 1600]  min samples split = [5,10,.., 25]  number of random features = [sqrt, None ] | 10 fold |

Supplementary Table 4: Clinical sample characteristics. Number of subjects with major depressive disorder (MDD) in each category.

|  | **Number of episodes** | | **Antidepressant (AD) use** | | **Age of Onset** | |
| --- | --- | --- | --- | --- | --- | --- |
| **Cohorts** | First episode | Recurrent episodes | no AD | with AD | Adolescent | Adults |
| AFFDIS | 3 | 30 | 1 | 32 | 61 | 18 |
| AMC | 20 | 22 | 50 | 0 | 24 | 24 |
| Barcelona | 22 | 40 | 4 | 58 | 13 | 49 |
| CARDIFF | 0 | 35 | 0 | 40 | 11 | 22 |
| CSAN | 14 | 46 | 31 | 29 | 0 | 0 |
| Calgary | 18 | 49 | 49 | 19 | 47 | 1 |
| DCHS | 0 | 0 | 0 | 0 | 0 | 0 |
| ETPB | 0 | 34 | 34 | 0 | 28 | 5 |
| Episca | 19 | 0 | 18 | 1 | 0 | 0 |
| FIDMAG | 11 | 22 | 4 | 30 | 6 | 27 |
| Groningen | 6 | 12 | 10 | 10 | 8 | 11 |
| Houston | 41 | 50 | 101 | 1 | 50 | 25 |
| Jena | 7 | 23 | 12 | 19 | 0 | 0 |
| LOND | 0 | 69 | 19 | 50 | 36 | 18 |
| MODECT | 0 | 33 | 32 | 10 | 0 | 0 |
| MPIP | 91 | 246 | 53 | 284 | 71 | 266 |
| Melbourne | 48 | 89 | 121 | 22 | 126 | 4 |
| Minnesota | 16 | 22 | 52 | 16 | 66 | 0 |
| Moraldilemma | 8 | 16 | 24 | 0 | 0 | 0 |
| NESDA | 67 | 87 | 98 | 56 | 78 | 76 |
| QTIM | 0 | 0 | 73 | 29 | 87 | 15 |
| SF | 32 | 34 | 75 | 0 | 60 | 0 |
| SHIP_S2 | 77 | 59 | 113 | 23 | 461 | 118 |
| SHIP_T0 | 113 | 197 | 257 | 53 | 974 | 255 |
| SanRaffaele | 1 | 44 | 2 | 42 | 7 | 37 |
| Singapore | 8 | 14 | 4 | 18 | 2 | 20 |
| Socat_dep | 19 | 60 | 41 | 38 | 121 | 28 |
| StanfFAA | 0 | 14 | 11 | 3 | 11 | 3 |
| StanfT1wAggr | 6 | 48 | 27 | 20 | 36 | 18 |
| TIGER | 29 | 20 | 29 | 20 | 49 | 0 |
| **All sites** | 676 | 1415 | 1345 | 923 | 2433 | 1040 |

Supplementary Table 5: Area under the curve (AUC) measured with cross-validation applied to the entire data set.

| **Splitting by Age/Sex** | | | | | | | | |
| --- | --- | --- | --- | --- | --- | --- | --- | --- |
|  | Cortical + Subcortical | | Cortical Thickness | | Cortical Surface area | | Subcortical Volume | |
|  | No ComBat | With ComBat | No ComBat | With ComBat | No ComBat | With ComBat | No ComBat | With ComBat |
| **Elastic Net** | 0.649 | 0.524 | 0.616 | 0.499 | 0.595 | 0.518 | 0.634 | 0.524 |
| **LASSO** | 0.650 | 0.524 | 0.617 | 0.500 | 0.597 | 0.521 | 0.634 | 0.524 |
| **Ridge** | 0.648 | 0.524 | 0.615 | 0.500 | 0.596 | 0.517 | 0.634 | 0.524 |
| **SVM PCA** | 0.681 | 0.541 | 0.636 | 0.527 | 0.601 | 0.516 | 0.663 | 0.519 |
| **SVM + ttest** | 0.667 | 0.527 | 0.619 | 0.530 | 0.589 | 0.528 | 0.656 | 0.523 |
| **SVM linear** | 0.654 | 0.485 | 0.614 | 0.500 | 0.598 | 0.509 | 0.635 | 0.512 |
| **SVM rbf** | 0.677 | 0.536 | 0.636 | 0.530 | 0.610 | 0.513 | 0.664 | 0.528 |
| **Random Forests** | 0.676 | 0.549 | 0.633 | 0.515 | 0.615 | 0.522 | 0.662 | 0.532 |
| **Splitting by Site** | | | | | | | | |
|  | Cortical + Subcortical | | Cortical Thickness | | Cortical Surface area | | Subcortical Volume | |
|  | No ComBat | With ComBat | No ComBat | With ComBat | No ComBat | With ComBat | No ComBat | With ComBat |
| **Elastic Net** | 0.524 | 0.522 | 0.502 | 0.487 | 0.505 | 0.519 | 0.524 | 0.522 |
| **LASSO** | 0.524 | 0.523 | 0.492 | 0.487 | 0.507 | 0.520 | 0.524 | 0.522 |
| **Ridge** | 0.524 | 0.522 | 0.497 | 0.487 | 0.505 | 0.518 | 0.524 | 0.522 |
| **SVM PCA** | 0.524 | 0.526 | 0.494 | 0.510 | 0.498 | 0.528 | 0.525 | 0.526 |
| **SVM + ttest** | 0.510 | 0.521 | 0.484 | 0.504 | 0.509 | 0.514 | 0.510 | 0.528 |
| **SVM linear** | 0.523 | 0.509 | 0.499 | 0.507 | 0.506 | 0.507 | 0.517 | 0.525 |
| **SVM rbf** | 0.522 | 0.521 | 0.492 | 0.504 | 0.496 | 0.513 | 0.516 | 0.520 |
| **Random Forests** | 0.519 | 0.526 | 0.496 | 0.501 | 0.496 | 0.523 | 0.529 | 0.514 |

Supplementary Table 6: Sensitivity measured with cross-validation applied to the entire data.

| **Splitting by Age/Sex** | | | | | | | | |
| --- | --- | --- | --- | --- | --- | --- | --- | --- |
|  | Cortical + Subcortical | | Cortical Thickness | | Cortical Surface area | | Subcortical Volume | |
|  | No ComBat | With ComBat | No ComBat | With ComBat | No ComBat | With ComBat | No ComBat | With ComBat |
| **Elastic Net** | 0.581 | 0.514 | 0.544 | 0.488 | 0.533 | 0.508 | 0.572 | 0.510 |
| **LASSO** | 0.587 | 0.515 | 0.546 | 0.479 | 0.532 | 0.506 | 0.575 | 0.505 |
| **Ridge** | 0.582 | 0.513 | 0.548 | 0.489 | 0.535 | 0.510 | 0.573 | 0.509 |
| **SVM PCA** | 0.561 | 0.450 | 0.532 | 0.392 | 0.473 | 0.489 | 0.555 | 0.481 |
| **SVM + ttest** | 0.571 | 0.503 | 0.471 | 0.452 | 0.370 | 0.470 | 0.557 | 0.494 |
| **SVM linear** | 0.563 | 0.509 | 0.476 | 0.478 | 0.490 | 0.516 | 0.574 | 0.525 |
| **SVM rbf** | 0.576 | 0.413 | 0.516 | 0.452 | 0.499 | 0.487 | 0.555 | 0.476 |
| **Random Forests** | 0.415 | 0.080 | 0.442 | 0.237 | 0.336 | 0.126 | 0.429 | 0.145 |
| **Splitting by Site** | | | | | | | | |
|  | Cortical + Subcortical | | Cortical Thickness | | Cortical Surface area | | Subcortical Volume | |
|  | No ComBat | With ComBat | No ComBat | With ComBat | No ComBat | With ComBat | No ComBat | With ComBat |
| **Elastic Net** | 0.509 | 0.506 | 0.492 | 0.484 | 0.443 | 0.510 | 0.524 | 0.510 |
| **LASSO** | 0.507 | 0.508 | 0.498 | 0.484 | 0.436 | 0.513 | 0.525 | 0.507 |
| **Ridge** | 0.509 | 0.506 | 0.495 | 0.485 | 0.440 | 0.507 | 0.524 | 0.510 |
| **SVM PCA** | 0.441 | 0.491 | 0.458 | 0.453 | 0.360 | 0.529 | 0.445 | 0.516 |
| **SVM + ttest** | 0.463 | 0.517 | 0.381 | 0.426 | 0.278 | 0.484 | 0.456 | 0.502 |
| **SVM linear** | 0.477 | 0.506 | 0.433 | 0.479 | 0.395 | 0.508 | 0.492 | 0.522 |
| **SVM rbf** | 0.455 | 0.467 | 0.403 | 0.426 | 0.379 | 0.513 | 0.451 | 0.508 |
| **Random Forests** | 0.254 | 0.102 | 0.394 | 0.218 | 0.138 | 0.139 | 0.296 | 0.154 |

Supplementary Table 7: Specificity measured with cross-validation applied to the entire data set.

| **Splitting by Age/Sex** | | | | | | | | |
| --- | --- | --- | --- | --- | --- | --- | --- | --- |
|  | Cortical + Subcortical | | Cortical Thickness | | Cortical Surface area | | Subcortical Volume | |
|  | No ComBat | With ComBat | No ComBat | With ComBat | No ComBat | With ComBat | No ComBat | With ComBat |
| **Elastic Net** | 0.637 | 0.532 | 0.623 | 0.513 | 0.604 | 0.526 | 0.614 | 0.531 |
| **LASSO** | 0.637 | 0.533 | 0.620 | 0.518 | 0.624 | 0.527 | 0.616 | 0.530 |
| **Ridge** | 0.637 | 0.533 | 0.622 | 0.507 | 0.612 | 0.520 | 0.614 | 0.531 |
| **SVM PCA** | 0.714 | 0.609 | 0.670 | 0.634 | 0.677 | 0.548 | 0.689 | 0.546 |
| **SVM + ttest** | 0.683 | 0.527 | 0.691 | 0.578 | 0.764 | 0.582 | 0.681 | 0.548 |
| **SVM linear** | 0.670 | 0.539 | 0.679 | 0.530 | 0.655 | 0.521 | 0.629 | 0.522 |
| **SVM rbf** | 0.703 | 0.636 | 0.684 | 0.578 | 0.656 | 0.533 | 0.682 | 0.550 |
| **Random Forests** | 0.811 | 0.934 | 0.744 | 0.791 | 0.811 | 0.893 | 0.794 | 0.877 |
| **Splitting by Site** | | | | | | | | |
|  | Cortical + Subcortical | | Cortical Thickness | | Cortical Surface area | | Subcortical Volume | |
|  | No ComBat | With ComBat | No ComBat | With ComBat | No ComBat | With ComBat | No ComBat | With ComBat |
| **Elastic Net** | 0.517 | 0.523 | 0.504 | 0.494 | 0.564 | 0.518 | 0.490 | 0.518 |
| **LASSO** | 0.518 | 0.525 | 0.484 | 0.494 | 0.580 | 0.514 | 0.488 | 0.517 |
| **Ridge** | 0.519 | 0.522 | 0.498 | 0.494 | 0.571 | 0.511 | 0.489 | 0.518 |
| **SVM PCA** | 0.612 | 0.550 | 0.546 | 0.571 | 0.649 | 0.520 | 0.595 | 0.525 |
| **SVM + ttest** | 0.541 | 0.508 | 0.593 | 0.571 | 0.736 | 0.533 | 0.564 | 0.552 |
| **SVM linear** | 0.546 | 0.533 | 0.563 | 0.510 | 0.603 | 0.505 | 0.519 | 0.520 |
| **SVM rbf** | 0.570 | 0.562 | 0.583 | 0.571 | 0.607 | 0.513 | 0.556 | 0.529 |
| **Random Forests** | 0.783 | 0.909 | 0.596 | 0.783 | 0.845 | 0.866 | 0.742 | 0.848 |

Supplementary Table 8: Balanced accuracy measured with cross-validation applied to the entire data set under different harmonization options.

|  | **Splitting by Age/Sex** | | | **Splitting by Site** | | |
| --- | --- | --- | --- | --- | --- | --- |
|  | ComBat | ComBat-GAM | CovBat | ComBat | ComBat-GAM | CovBat |
| **Elastic Net** | 0.523 | 0.522 | 0.517 | 0.514 | 0.515 | 0.514 |
| **LASSO** | 0.524 | 0.523 | 0.517 | 0.517 | 0.513 | 0.514 |
| **Ridge** | 0.523 | 0.519 | 0.518 | 0.514 | 0.516 | 0.514 |
| **SVM PCA** | 0.529 | 0.521 | 0.523 | 0.520 | 0.520 | 0.528 |
| **SVM + ttest** | 0.515 | 0.503 | 0.513 | 0.512 | 0.505 | 0.512 |
| **SVM linear** | 0.524 | 0.526 | 0.521 | 0.519 | 0.520 | 0.518 |
| **SVM rbf** | 0.525 | 0.522 | 0.522 | 0.515 | 0.519 | 0.512 |

Supplementary Table 9: Balanced accuracy of support vector machines (SVM) with linear kernel without feature selection trained and validated with leave-one-site-out cross-validation. More extreme values of balanced accuracy were obtained for cohorts containing no healthy subjects. Note that ComBat brings these values closer to average across all cohorts.

| **Cohort** | Balanced Accuracy  **No ComBat** | Balanced Accuracy  **With ComBat** | **Ratio MDD/HC** |
| --- | --- | --- | --- |
| **SHIP T0** | 0.503017 | 0.50513 | 0.3373232 |
| **SHIP S2** | 0.474821 | 0.516075 | 0.3069977 |
| **StanfT1wAggr** | 0.535866 | 0.476998 | 0.9491525 |
| **Minnesota** | 0.532143 | 0.521429 | 1.75 |
| **CSAN** | 0.489966 | 0.531973 | 1.2244898 |
| **Jena** | 0.498268 | 0.549567 | 0.3896104 |
| **Calgary** | 0.522727 | 0.553147 | 1.0576923 |
| **Barc** | 0.489415 | 0.467742 | 1.9375 |
| **DCHS** | 0.514117 | 0.607013 | 0.295082 |
| **AFFDIS** | 0.496377 | 0.455534 | 0.7173913 |
| **Moraldilemma** | 0.67663 | 0.586051 | 0.5217391 |
| **FIDMAG** | 0.487815 | 0.535714 | 1.0294118 |
| **MPIP** | 0.532092 | 0.533309 | 1.5971564 |
| **ETPB** | 0.561086 | 0.529412 | 1.3076923 |
| **TIGER** | 0.569573 | 0.517625 | 4.4545455 |
| **AMC** | 0.313726 | 0.431373 | N/A |
| **Episca(Leiden)** | 0.563158 | 0.513158 | 0.6333333 |
| **SanRaffaele** | 0.911111 | 0.644444 | N/A |
| **MODECT** | 0.785714 | 0.309524 | N/A |
| **Gron** | 0.519048 | 0.585714 | 0.952381 |
| **CARDIFF** | 0.85 | 0.5 | N/A |
| **Singapore** | 0.434659 | 0.400568 | 1.375 |
| **StanfFAA** | 0.468254 | 0.373016 | 0.7777778 |
| **QTIM** | 0.506663 | 0.509183 | 0.3591549 |
| **Houst** | 0.491057 | 0.477512 | 0.5591398 |
| **Melb** | 0.568216 | 0.546551 | 1.4019608 |
| **NESDA** | 0.52952 | 0.533417 | 2.3692308 |
| **Socat_dep** | 0.527722 | 0.547468 | 0.79 |
| **UCSF** | 0.522197 | 0.506061 | 0.8522727 |
| **LOND** | 0.531005 | 0.609527 | 1.1311475 |
| **ALL SITES** | 0.513 | 0.515 | 0.74 |

Supplementary Table 10: Balanced accuracy of support vector machines (SVM) with linear kernel without feature selection trained and validated using single site data with 10-fold cross-validation. More extreme values in balanced accuracies are observed in smaller cohorts, while bigger cohorts tend to produce accuracies close to 50-55%. MDD/HC ratio and balanced accuracy for all sites are presented as average in the last line.

| Cohorts | Number of Subjects | Ratio MDD/HC | Balanced Accuracy |
| --- | --- | --- | --- |
| AFFDIS | 79 | 0.717 | 0.477 |
| AMC | 51 | N/A | N/A |
| Barc | 94 | 1.938 | 0.593 |
| Calgary | 107 | 1.058 | 0.568 |
| CARDIFF | 40 | N/A | N/A |
| CSAN | 109 | 1.224 | 0.584 |
| DCHS | 79 | 0.295 | 0.613 |
| Episca | 49 | 0.633 | 0.423 |
| ETPB | 60 | 1.308 | 0.446 |
| FIDMAG | 69 | 1.029 | 0.662 |
| Gron | 41 | 0.952 | 0.540 |
| Houst | 290 | 0.559 | 0.550 |
| Jena | 107 | 0.390 | 0.585 |
| LOND | 130 | 1.131 | 0.484 |
| Melb | 245 | 1.402 | 0.520 |
| Minnesota | 110 | 1.750 | 0.583 |
| MODECT | 42 | N/A | N/A |
| Moraldilemma | 70 | 0.522 | 0.706 |
| MPIP | 548 | 1.597 | 0.601 |
| NESDA | 219 | 2.369 | 0.488 |
| QTIM | 386 | 0.359 | 0.464 |
| SanRaffaele | 45 | N/A | N/A |
| SF | 163 | 0.852 | 0.523 |
| SHIP_S2 | 579 | 0.307 | 0.554 |
| SHIP_T0 | 1229 | 0.337 | 0.495 |
| Singapore | 38 | 1.375 | 0.725 |
| Socat_dep | 179 | 0.790 | 0.557 |
| StanfFAA | 32 | 0.778 | 0.584 |
| StanfT1wAggr | 115 | 0.949 | 0.514 |
| TIGER | 60 | 4.455 | 0.558 |
| All sites | 5265 | 1.118 | 0.553 |


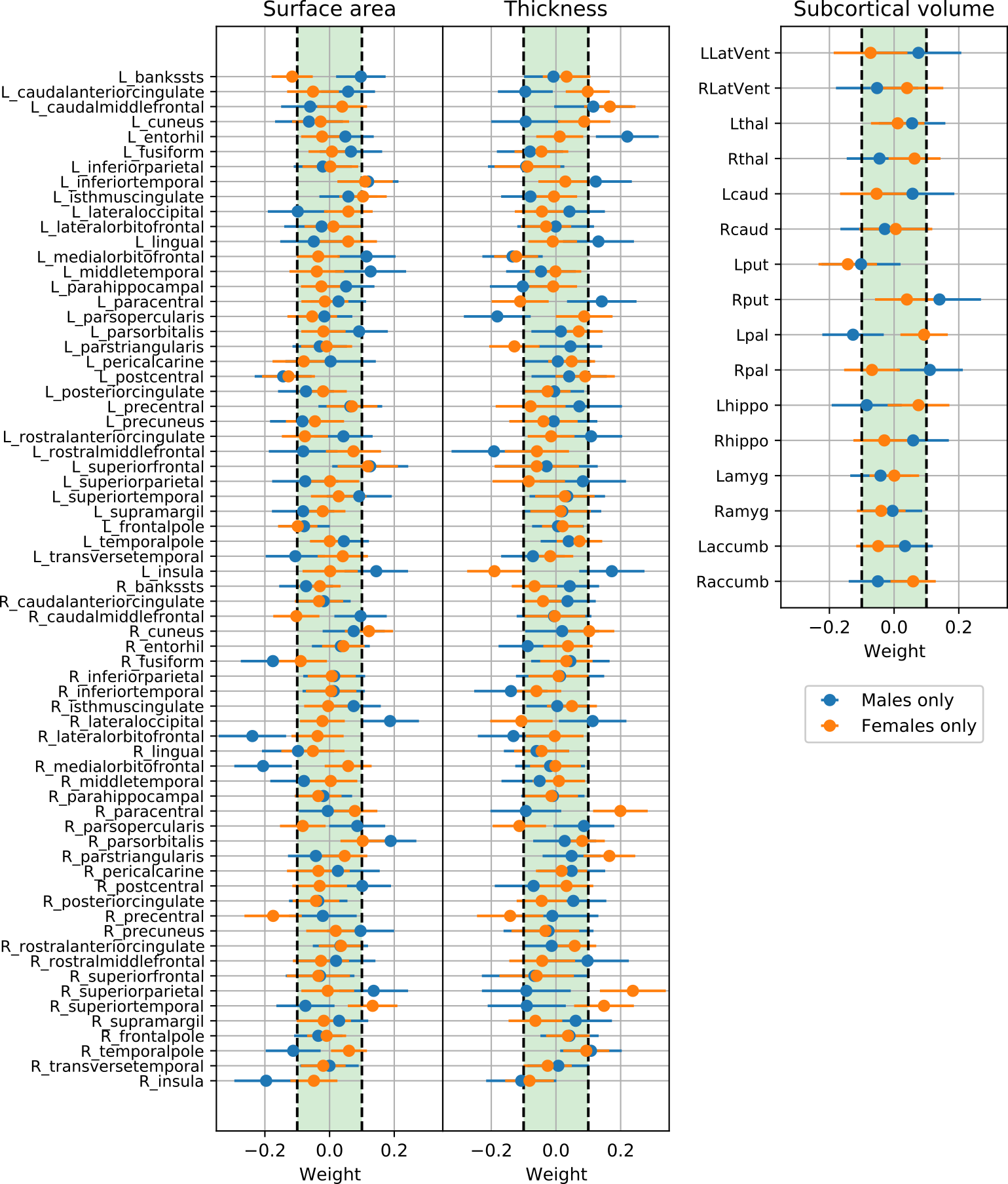


Supplementary Figure 1: Weights of SVM with linear kernel applied on stratified data by sex (no feature selection, with ComBat). The horizontal bars indicate the 95% confidence interval calculated using percentile method via bootstrapping.


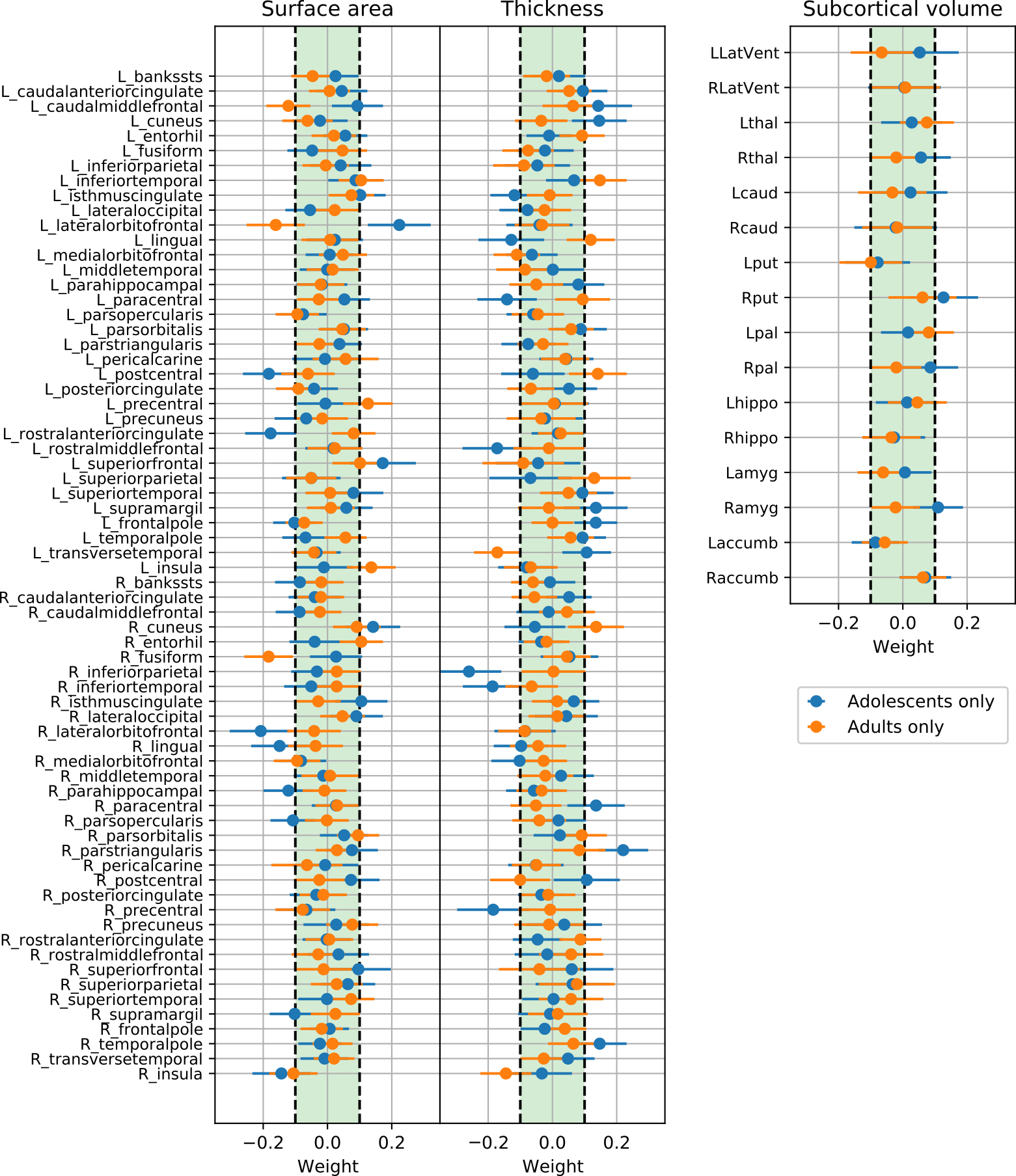


Supplementary Figure 2: Weights of SVM with linear kernel applied on stratified data by age of onset (no feature selection ,with ComBat). The horizontal bars indicate the 95% confidence interval calculated using percentile method via bootstrapping.


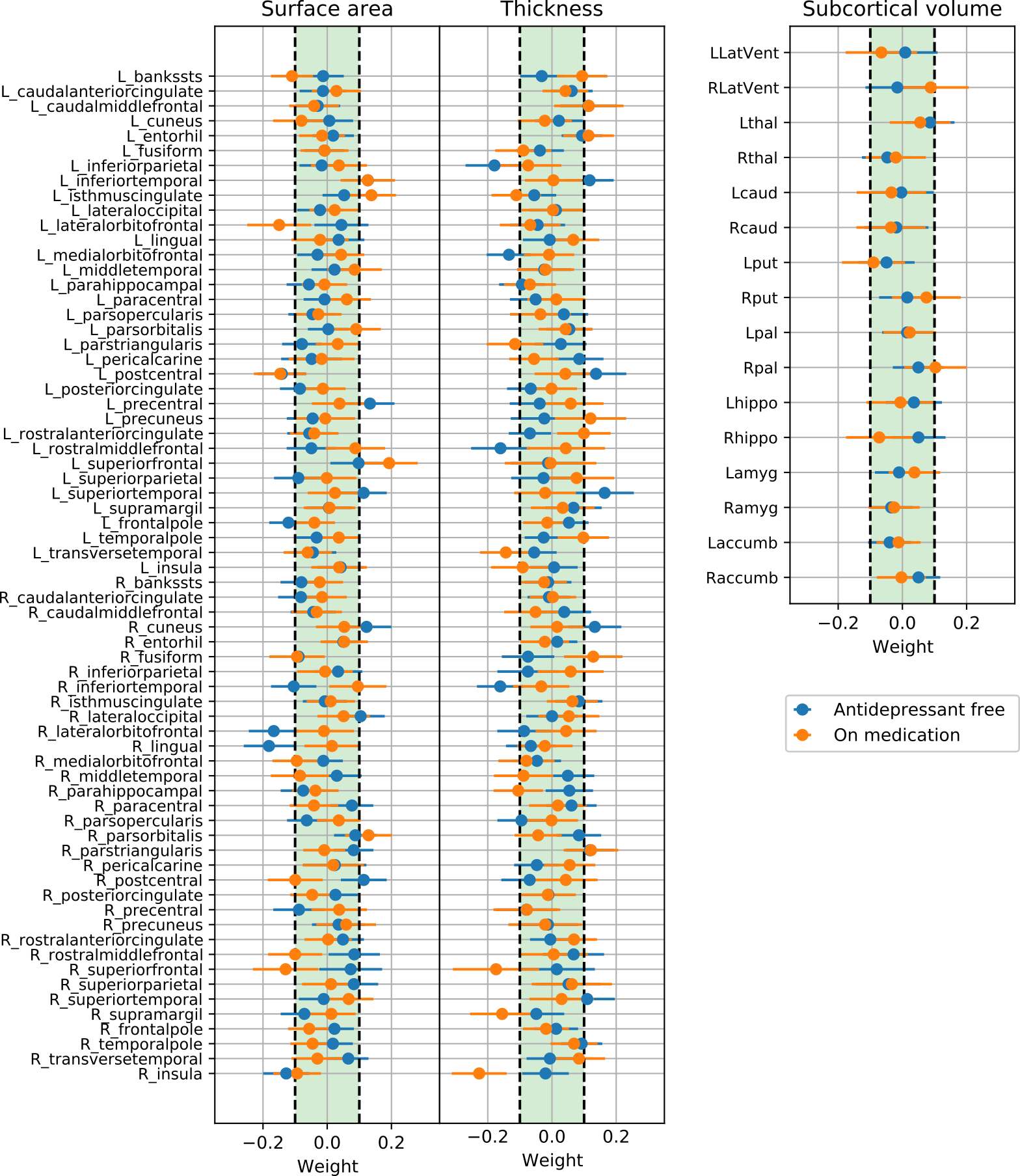


Supplementary Figure 3: Weights of SVM with linear kernel applied on stratified data by use of antidepressant medication (no feature selection, with ComBat). The horizontal bars indicate the 95% confidence interval calculated using percentile method via bootstrapping.


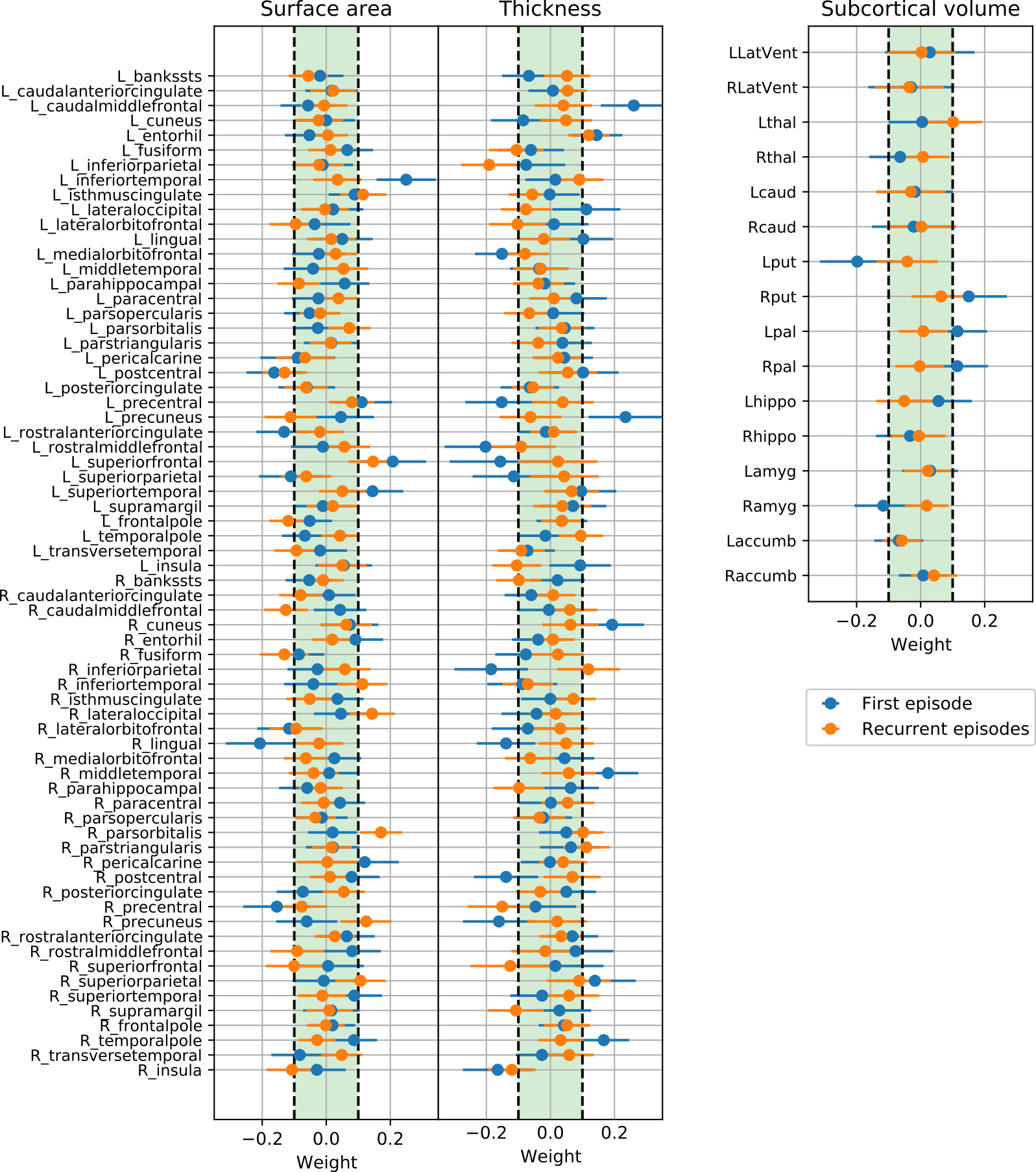


Supplementary Figure 4: Weights of SVM with linear kernel applied on stratified data by number of episodes (no feature selection, with ComBat). The horizontal bars indicate the 95% confidence interval calculated using percentile method via bootstrapping.

*CV splitting strategies*

We wrote our own scripts in python to perform both splitting strategies, which were different in Splitting by Site and Splitting by Age/Sex. The codes are publicly available (<https://github.com/vlbl/Splitting-by-Site>). In Splitting by Site, we balanced the number of subjects across folds as much as possible. Conversely, in Splitting by Age/Sex, we balanced the number of subjects across folds. It was achieved by assigning every subject to the fold, which leads to more even age/sex distribution (by comparing the mean of all folds to the mean of the fold when subject is added) across the folds. This process is repeated for every site separately. Thus, each fold contains almost equal number of subjects per site.

*Harmonization methods*

We harmonized individual cortical and subcortical features by implementing the well-established statistical harmonization algorithm, ComBat ^3^. Its purpose was to adjust Location (mean) and Scale (variation) (L/S) of all features of the data collected from different cohorts by preserving the influence of biologically-significant factors of interest in the features. Additionally, it is assumed that the site effect is not independent across cortical and subcortical features and it uses empirical Bayes for site effect estimation. Subsequently, the cortical and subcortical features would be standardized, while the site effect would be removed. ComBat assumes that the data $Y_{i,j,k}$ for ROI *k*, site *i* and subject *j* can be represented by the following model:

$$\begin{aligned} Y_{ijk}= \alpha_{k}+X_{ij}\beta_{k}+ \gamma_{ik}+ \delta_{ik}\varepsilon_{ijk} \#\left( 1 \right) \end{aligned}$$

Where $\alpha_{k}$ is an overall ROI value, $X$is a design matrix where $X_{ij}$j is a vector containing site affiliation and controlled covariates of participant j in site i.. In our case these are age, sex and ICV. $\beta_{k}$is the vector of regression coefficients corresponding to $X_{ij}$, $\gamma_{ik}$ and $\delta_{ik}$ correspond to additive and multiplicative site effect and $\varepsilon_{ijk}$ is an error term assumed to follow normal distribution with mean zero and variance $\sigma_{k}^{2}$ .After parameter estimation in the model above, the standardized data ${Y^{*}}_{ijk}$ can be calculated as follows:

$$\begin{aligned} {Y^{*}}_{ijk}=\frac{Y_{ijk}- \hat{\alpha}_{k}-X_{ij}\hat{\beta}_{k}- \hat{\gamma}_{ik}}{\hat{\delta}_{ik}} +\hat{\alpha}_{k}+ X_{ij}\hat{\beta}_{k}\#\left( 2 \right) \end{aligned}$$

where $\hat{\alpha}_{k}$, $\hat{\beta}_{k},\hat{\gamma}_{ik}$ and $\hat{\delta}_{k}$ are estimated ComBat parameters. Additionally, it is assumed that the site effect is not independent across cortical and subcortical features.

All parameter estimations, which includes estimates of $\hat{\alpha}_{k}$, $\hat{\beta}_{k},\hat{\gamma}_{ik}$ and $\hat{\delta}_{k}$ , should be computed only on the training set, i.e. 9 CV folds, to avoid non-independence of the training and test data, also known as data leakage. After parameter estimations and training of the ML algorithm were complete, the calculated parameters were used to adjust the test data and the performance of the trained classification algorithm measured on the test set represented by the remaining CV fold. These parameters were directly used for adjusting data from unseen subjects from the test set only if these subjects belong to the same cohorts as in the training set. This scenario corresponds to Splitting by Age/Sex strategy as every CV fold contains subjects from all cohorts.

In Splitting by Site strategy, subjects from one cohort are included only in one CV fold, thus the direct usage of estimated ComBat parameters on the test set is imprudent. Here we adapted the approach of a reference batch adjustment ^4^, which constitutes fixing a reference sites, while other sites are adjusted to the mean and variance of the reference site according to the following equation:

$$\begin{aligned} {Y^{*}}_{ijkr}=\frac{Y_{ijk}- \hat{\alpha}_{rk}-X_{ij}\hat{\beta}_{kr}- \hat{\gamma}_{ikr}}{\hat{\delta}_{ikr}} +\hat{\alpha}_{kr}+ X_{ij}\hat{\beta}_{kr}\#\left( 3 \right) \end{aligned}$$

where $\alpha_{kr}$, $\beta_{kr}$ correspond to coefficients estimated on the reference site *r.* Additionally, $\gamma_{ikr}$, $\delta_{ikr}$ represent additive and multiplicative differences between site i and r. In our case, the test set was adjusted to a unified batch made by integrating all cohorts from the training set and adjusting to common mean and variance by the ComBat, which allowed to harmonize unseen cohorts without data leakage from the training set to the test set (Supplementary Figure 5).

This framework was additionally extended to include non-linear preservation of the covariates by substituting $X_{ij}\beta_{k}$with a Generalized Additive Model (ComBat-GAM) ^5^, allowing nonlinear age trends to be preserved during the harmonization step. Furthermore, we considered a CovBat model, which assumes an additional covariance site effect alongside with mean and variance corrections ^6^.We tested ComBat’s harmonization ability to remove the site effect from the full data by training cortical and subcortical features via SVM with a linear kernel to predict the site. This was mostly tested in cases where the number of sites was below 10 ^5,7,8^, so it is relevant in the context of our current analysis. We used the Splitting by Age/Sex strategy, since to predict site information from the test folds, this information should be presented in the training folds - and this would not be possible in the Splitting by Site strategy. The balanced accuracy was 0.854 before applying ComBat without correction for age, sex and ICV. Such a high performance indicated a strong site effect presented in the data, which may interfere with the main MDD vs HC classification task. The classification balanced accuracy dropped substantially to 0.031 after applying ComBat, indicating the significant removal of site-related information in cortical and subcortical features. Such a low accuracy comes from the fact that with ComBat site-related information seemed to be removed from the data, resulting in SVM always predicting SHIP_T0 – the biggest cohort. By assessing the confusion matrices we could evidence that - without the harmonization step - the classification algorithm was able to predict site affiliation of the subject successfully, except of sites coming from the same research group e.g., MPIP (two cohorts) and in case of SHIP_T0-SHIP_S2 and Melbourne-MoralDilemma pairs. As an illustration, an example of a feature being harmonized via ComBat may be seen in Supplementary Figure 6.

To further investigate whether differences across sites were mainly driven by irregular age and sex distributions, we repeated the classification task by also regressing out age and sex from the features. This resulted in 0.816 and 0.031 balanced accuracies for predicting site without and with ComBat harmonization respectively. By comparing the results with and without this residualization step, we could infer that the classification performance in differentiating sites only minorly came from age and sex distribution as it remained very similar to the previous classification results. To see if the site effect was due to the differences in scanners and scan acquisition protocols between cohorts, we trained SVM to predict scanner type from cortical and subcortical features. The resulting accuracy was 0.875, even higher than only site prediction. This hints to the site effect being primarily caused by differences in acquisition equipment across sites.


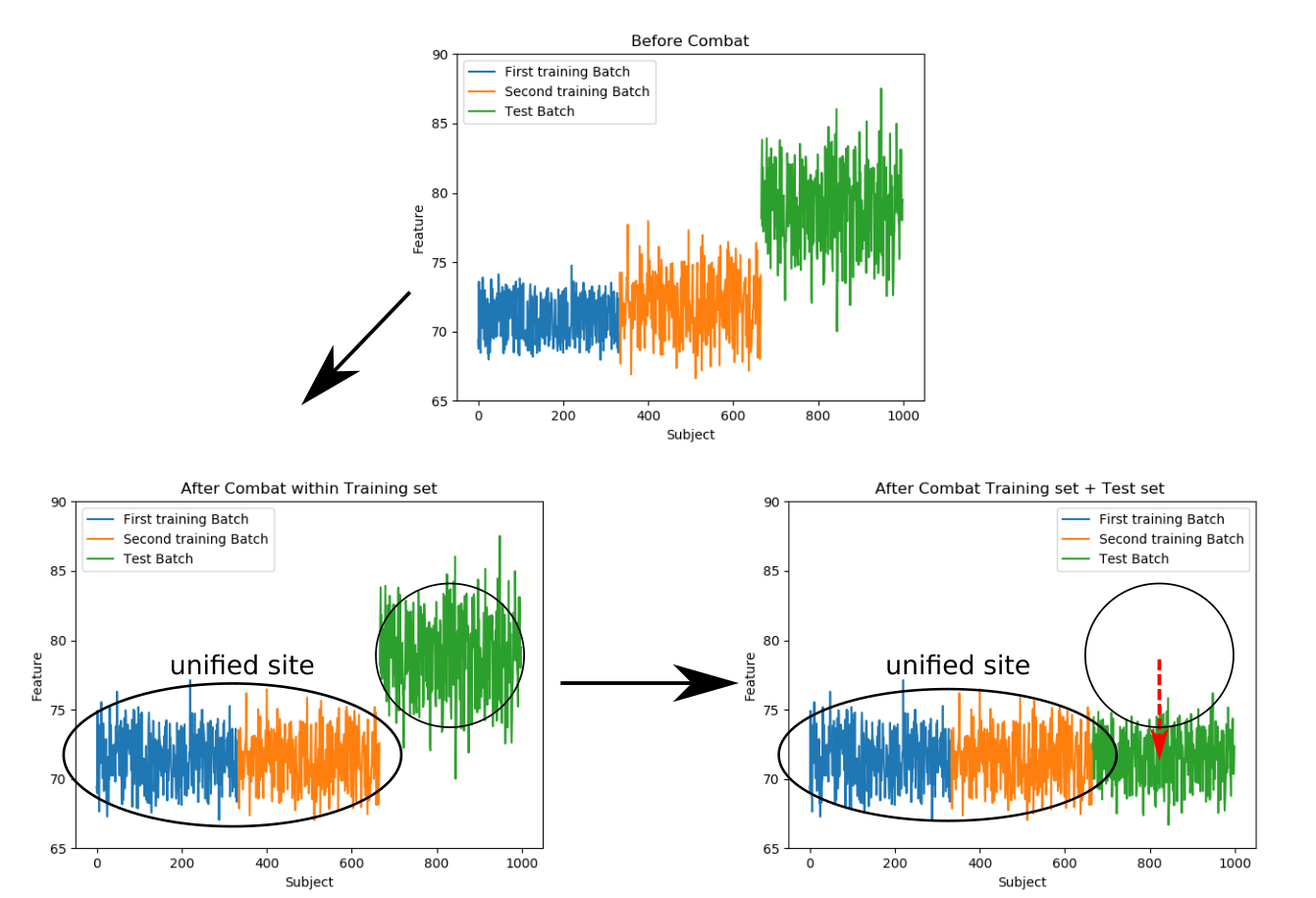


Supplementary Figure 5: Test set adjustment to the unified site. After ComBat is applied on the training set, all training sites are adjusted so that their residuals (after fitting covariates) have the same mean and variance, which we unify to build a unified site used for the classification training. After the training is complete, test set is harmonized to the fixed unified site allowing trained model to be evaluated on the test set.


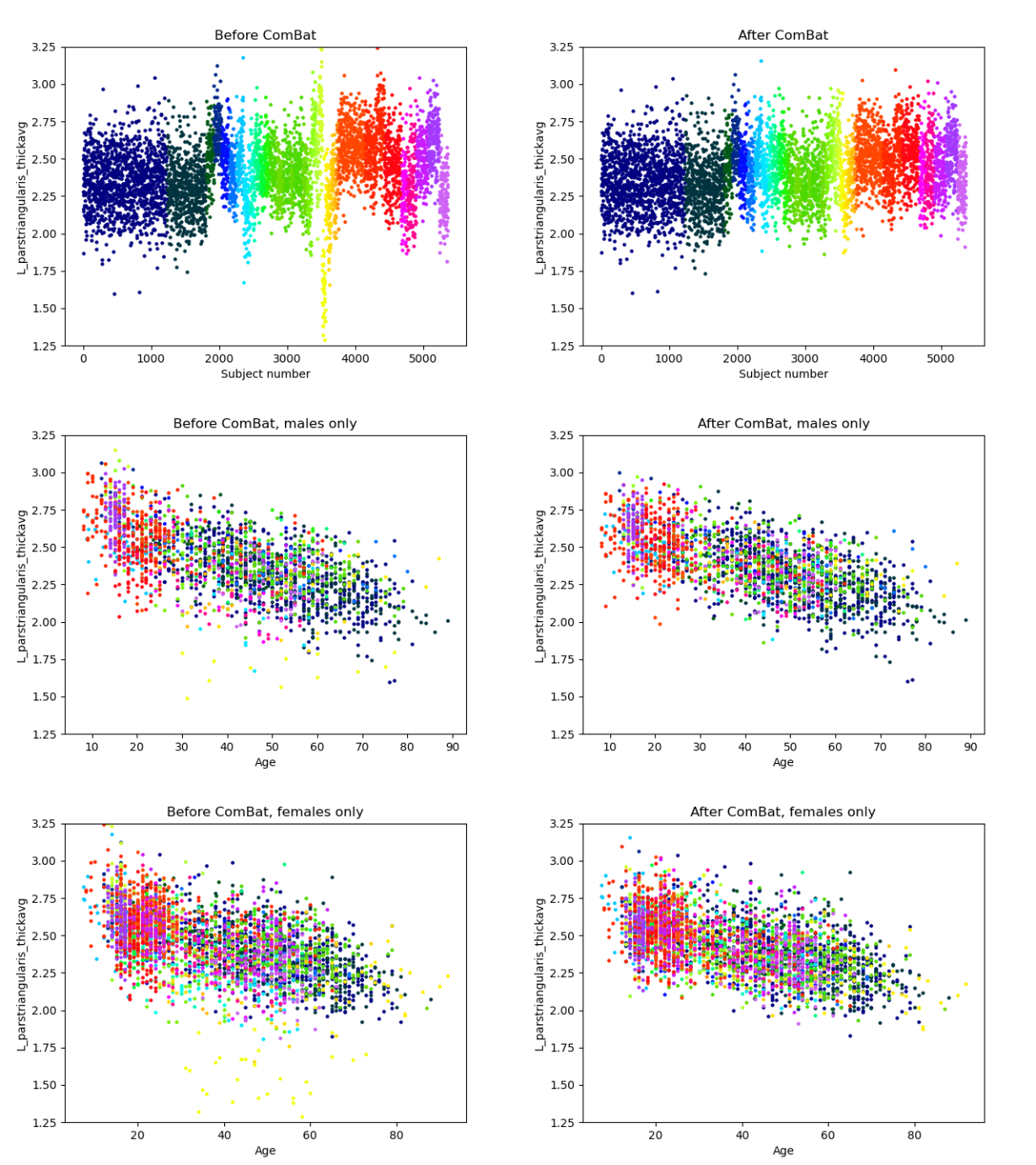


Supplementary Figure 6**:** An example of site effect removal by ComBat for left pars opercularis thickness. Color corresponds to the site affiliation. While the differences between sites are reduced, remaining differences correspond to age- and sex-related differences between cohorts (middle and bottom).

**References**

1. Hsu, C., Chang, C. & Lin, C.-J. A Practical Guide to Support Vector Classification Chih-Wei Hsu, Chih-Chung Chang, and Chih-Jen Lin. (2003).

2. Liaw, A. Classification and Regression by randomForest. *R News* **2**, 18–22 (2002).

3. Johnson, W. E., Li, C. & Rabinovic, A. Adjusting batch effects in microarray expression data using empirical Bayes methods. *Biostatistics* **8**, 118–127 (2007).

4. Zhang, Y., Jenkins, D. F., Manimaran, S. & Johnson, W. E. Alternative empirical Bayes models for adjusting for batch effects in genomic studies. *BMC Bioinformatics* **19**, 262 (2018).

5. Pomponio, R. *et al.* Harmonization of large MRI datasets for the analysis of brain imaging patterns throughout the lifespan. *Neuroimage* **208**, 116450 (2020).

6. Chen, A. A. *et al.* Removal of Scanner Effects in Covariance Improves Multivariate Pattern Analysis in Neuroimaging Data. *bioRxiv* 858415 (2020) doi:10.1101/858415.

7. Garcia-Dias, R. *et al.* Neuroharmony: A new tool for harmonizing volumetric MRI data from unseen scanners. *Neuroimage* **220**, 117127 (2020).

8. Radua, J. *et al.* Increased power by harmonizing structural MRI site differences with the ComBat batch adjustment method in ENIGMA. *NeuroImage* **218**, (2020).
